# Nuciferine Ameliorates Ovariectomy-Induced Bone Loss via TFEB-Mediated Autophagy-Lysosomal Pathway

**DOI:** 10.3390/biom16091370

**Published:** 2026-09-21

**Authors:** Beilei Shi, Ze Gao, Shih-Yu Chen, Jianbing Chen, Liangxu Wei, Wen Fan, Zhiqian Yang, Ying Zhang, Yan Cui, Xiaofeng Zhu

**Affiliations:** 1 School of Traditional Chinese Medicine, Jinan University, Guangzhou 510630, China; beilei@stu2024.jnu.edu.cn (B.S.); ensyuu29@gmail.com (S.-Y.C.); m15393110537@163.com (J.C.); weilangxu@stu2025.jnu.edu.cn (L.W.); fanww1999@stu2022.jnu.edu.cn (W.F.); zhangying0809@stu2025.jnu.edu.cn (Y.Z.); 2 The First Affiliated Hospital of Jinan University, Guangzhou 510630, China; gaoze@stu2024.jnu.edu.cn (Z.G.); zqykennis@stu2024.jnu.edu.cn (Z.Y.); 3 Guangdong Provincial Key Laboratory of Traditional Chinese Medicine Informatization, Guangzhou 510632, China

**Keywords:** nuciferine, osteoporosis, osteogenic differentiation, transcription factor EB, autophagy-lysosomal pathway

## Abstract

**Background:** Postmenopausal osteoporosis is a common metabolic bone disease that leads to reduced bone formation and increased fracture risk. Nuciferine, an aporphine alkaloid from the lotus leaf, can regulate the TFEB-mediated autophagy-lysosomal pathway. This pathway is critical for osteogenic differentiation. However, it remains unclear whether nuciferine promotes bone formation and alleviates bone loss through this pathway. We therefore hypothesized that nuciferine activates the TFEB-mediated autophagy-lysosomal pathway to promote osteogenic differentiation, thereby counteracting estrogen deficiency-induced bone loss. **Methods:** We established an OVX rat model (6-month-old females, 24.0 ± 1.0 weeks, *n* = 10/group), treated the rats with nuciferine via oral gavage for 12 weeks, and used rat BMSCs for mechanistic studies in vitro. **Results:** Nuciferine significantly increased bone mineral density (BMD) and improved bone microarchitecture in OVX rats; upregulated serum levels of the bone turnover markers bone-specific alkaline phosphatase (BALP), procollagen type I N-terminal propeptide (PINP) and osteocalcin (BGP); and downregulated serum tartrate-resistant acid phosphatase (TRAP), as measured via ELISA. Nuciferine also restored the expression of osterix (OSX) and transcription factor EB (TFEB) in bone tissue, as detected via TSA staining. Furthermore, it increased the microtubule-associated protein 1 light chain 3 (LC3)-II/LC3-I ratio and decreased p62 expression, as determined through Western blotting, indicating the activation of autophagy. In vitro, nuciferine promoted osteogenic differentiation and TFEB nuclear translocation in BMSCs, whereas TFEB silencing suppressed both, an effect partially reversed by nuciferine, as shown via ALP/ARS staining, Western blotting, RT-qPCR, and immunofluorescence. **Conclusions:** In conclusion, nuciferine ameliorates bone loss in OVX rats and promotes the osteogenic differentiation of BMSCs. Activation of the TFEB-mediated autophagy-lysosomal pathway is a key mechanism underlying its improvement of bone metabolism.

## 1. Introduction

Osteoporosis (OP) is a common chronic metabolic bone disease. It features a loss of bone mass and a decline in bone microarchitecture [1], leading to weaker bones and a much higher chance of fractures [2,3]. OP is characterized by high incidence, high disability, high mortality, and low public awareness, with a particularly high incidence in postmenopausal women, placing a major strain on personal health and societal medical resources [4]. The occurrence of osteoporosis is tightly linked to an imbalance in bone remodeling, the core pathological mechanism of which lies in enhanced bone resorption accompanied by insufficient osteogenic function. Impaired osteogenesis caused by postmenopausal estrogen deficiency and aging is an important contributor to defective bone formation and bone loss [5]. Currently, the most widely used bone-forming agents are parathyroid hormone (PTH), its analogs, and anti-sclerostin monoclonal antibodies, supplemented by active vitamin D and vitamin K [6]. The representative agent teriparatide is expensive and has a limited course of treatment; after discontinuation, antiresorptive drugs are required to maintain its effect, and it is associated with hypercalcemia, hypercalciuria, urolithiasis, and the risk of osteosarcoma [7]. Romosozumab has a stronger bone-forming effect but can likewise only be used for a short period, is accompanied by the risk of cardiovascular events, and may cause hypocalcemia [8]. Therefore, identifying active molecules that can effectively promote osteogenic differentiation, improve defective bone formation, and have favorable safety profiles remains an important issue to be addressed in the management of osteoporosis.

An increasing number of studies suggest that the autophagy-lysosomal system is not only an important quality control mechanism for maintaining intracellular homeostasis but also a key link in regulating osteoblast differentiation and bone formation [9,10]. TFEB (transcription factor EB), a central controller of the autophagy-lysosomal pathway, maintains normal osteoblast function by promoting lysosomal biogenesis, enhancing autophagic flux, and coordinating cellular energy metabolism [11,12]. Evidence has indicated that TFEB activation is closely associated with enhanced osteogenic differentiation and that it functions to stimulate bone formation while preserving bone homeostasis by alleviating cellular stress injury and metabolic imbalance [13]. These findings suggest that, in the context of postmenopausal osteoporosis (PMOP), targeting the TFEB-mediated autophagy-lysosomal pathway may be an important approach for ameliorating impaired osteogenic capacity.

The lotus leaf, the dried leaf of Nelumbo nucifera Gaertn. (family Nymphaeaceae), has both medicinal and dietary value and is traditionally applied to clearing summer heat and resolving dampness, raising clear yang, and cooling blood to arrest bleeding [14]. Modern pharmacological research has revealed that the lotus leaf harbors multiple classes of bioactive compounds, including alkaloids, flavonoids, and polyphenols [15,16]. Among them, nuciferine (Nue) is one of the representative aporphine alkaloids, displaying biological properties, such as anti-inflammatory, antioxidant, lipid-regulating, and metabolism-improving activities [17,18]. As studies on the regulation of bone metabolism have advanced, the potential value of Nue in bone protection has gradually attracted attention. Existing studies show that Nue not only suppresses osteoclast formation and reduces bone resorption but also enhances type H vessel formation in bone, thereby contributing favorably to bone homeostasis [19]. In certain metabolic diseases, Nue is known to regulate the TFEB-mediated autophagy-lysosomal pathway [20]. However, whether Nue affects osteogenic differentiation by regulating the TFEB-mediated autophagy-lysosomal pathway and thereby ameliorates estrogen deficiency-induced bone loss still lacks direct evidence.

Given the important role of this mechanism in osteogenic differentiation and in the development of osteoporosis, we hypothesized that Nue may upregulate TFEB expression and promote its nuclear translocation, thereby activating the autophagy-lysosomal pathway to promote osteogenic differentiation and ameliorating estrogen deficiency-mediated bone loss. Based on this hypothesis, this study employed an OVX rat model of osteoporosis and in vitro osteogenic differentiation experiments to comprehensively examine the interventional effect of Nue on bone loss and its related molecular mechanisms. The aim of the study was to provide experimental support for the use of Nue in managing osteoporosis and to identify a new candidate molecule for anti-osteoporotic strategies targeting the TFEB-autophagy-lysosomal axis.

## 2. Materials and Methods

### 2.1. Reagents and Drugs

Nuciferine (T22559), chloroquine (T8689), and rapamycin (T1537) were obtained from Shanghai TopScience Biotechnology Co., Ltd. (Shanghai, China). Alendronate sodium (HJ20160100) was obtained from Organon (Shanghai, China). Antibodies and other reagents are listed in Supplementary Table S.1.

### 2.2. Cell Culture

Rat BMSCs were obtained from the Cell Bank of the Chinese Academy of Sciences (CAS, Shanghai, China).

### 2.3. Cell Viability Assay (CCK-8)

BMSCs were seeded into 96-well plates at 3 × 10^3^ cells per well in 100 µL of complete medium and allowed to adhere for 12 h at 37 °C in a 5% CO_2_ incubator. After attachment, the medium was replaced with fresh medium containing various concentrations of nuciferine (0, 0.1, 1, 10, 20, 40, 60, 80, and 100 μmol/L), with 5 replicate wells per concentration. After 3 or 5 days of culture, 10 µL of CCK 8 solution (Dojindo, Kumamoto, Japan; CK04) was added to each well, and the plates were incubated for 2 h at 37 °C in the dark. Absorbance was then recorded at 450 nm using a microplate reader (BioTek Epoch; Agilent Technologies, Santa Clara, CA, USA).

Cell viability was calculated as follows:

Cell viability (%) = [(OD(treatment) − OD(blank))/(OD(control) − OD(blank))] × 100%

where OD(treatment) represents the absorbance of nuciferine-treated wells, OD(control) represents the absorbance of cells cultured without drug treatment, and OD(blank) represents the background absorbance of wells containing only medium and CCK 8 solution. Each experiment was independently repeated at least three times.

### 2.4. Animals and Drug Administration

Three-month-old SPF-grade female Sprague–Dawley (SD) rats, weighing 250 ± 20 g, were purchased from Guangdong Vital River Laboratory Animal Technology Co., Ltd. (Foshan, Guangdong, China; license No. SCXK (Yue) 2022-0063). All animals were housed in the SPF-grade facility of the Laboratory Animal Center of Jinan University with free access to food and water, under conditions of 23 ± 2 °C, 45–50% relative humidity, and a 12 h light/dark cycle. The environment was kept quiet and well ventilated, and the bedding was changed two to three times per week. After one week of acclimation, the rats were raised to 6 months of age and then randomly divided into a sham-operated group (Sham), an ovariectomized group (OVX), an alendronate sodium group (ALE), a high-dose nuciferine group (Nue-H, 40 mg/kg), and a low-dose nuciferine group (Nue-L, 20 mg/kg), with 10 rats in each group. In the Sham group, the abdomen was opened and only a small amount of surrounding adipose tissue was excised, with both ovaries preserved. Rats in the other groups underwent bilateral ovariectomy to establish the osteoporosis model.

Ovariectomy (OVX) was performed as described previously [21]. Briefly, rats were fasted for 12 h with free access to water before surgery and weighed before anesthesia. Anesthesia was induced by intraperitoneal injection of 2% pentobarbital sodium at 0.2 mL/100 g, and adequate anesthesia was confirmed by the disappearance of the corneal reflex and pain response. After anesthesia, rats were fixed in the supine position, and the surgical area was shaved and routinely disinfected with iodophor and alcohol. Longitudinal incisions of approximately 2 cm were made approximately 1.5 cm below the bilateral costal arches, and the subcutaneous tissue and muscle layers were separated layer by layer to enter the abdominal cavity. After exposure and localization of the ovaries, the periovarian blood vessels and fallopian tubes were ligated, and both ovaries were removed. The peritoneum, muscle, and skin were sutured layer by layer, and the incisions were disinfected with 75% medical alcohol. The Sham group underwent the same procedure, except that only a small amount of adipose tissue was excised without ovariectomy. Penicillin was injected for 3 consecutive days after surgery to prevent infection.

One week after modeling, the successfully modeled SD rats were randomly divided into the model group (OVX), the low-dose nuciferine group (Nue-L, 20 mg/kg), the high-dose nuciferine group (Nue-H, 40 mg/kg), and the positive control group (ALE), with an additional sham-operated group (Sham). All treatments were given daily by gavage for 12 weeks [22], by which time the rats had reached 9 months of age. Outcome assessments were performed in a blinded manner. Nuciferine was prepared as a suspension with 0.5% sodium carboxymethyl cellulose (CMC-Na) as the solvent, and the model and Sham groups received an equal volume of 0.5% CMC-Na. Body weight was measured weekly to adjust the dosage. All animal experiments were approved by the Animal Ethics Committee of Jinan University (approval no. 20230307-01; approval date: 7 March 2023) and were performed in accordance with the relevant regulations on the administration of laboratory animals. 

### 2.5. Micro-CT Analysis

Micro-CT scanning of rat femurs was performed on the trabecular ROI using a Hiscan XM system (80 kV/100 µA; exposure 50 ms; voxel 25 µm; rotation 0.5°). Three-dimensional reconstruction and quantitative analysis were conducted using Hiscan Reconstruct and Analyzer software (V3.0; Hiscan, Suzhou, China).

### 2.6. Serum Estradiol and Bone Turnover Marker Assays

Blood samples were collected from the abdominal aorta of anesthetized rats, placed on ice, and centrifuged (4 °C, 3000 rpm, 20 min). Serum levels of estradiol (E2), bone-specific alkaline phosphatase (BALP), osteocalcin (BGP), tartrate-resistant acid phosphatase (TRAP), and procollagen type I N-terminal propeptide (PINP) were measured using commercial enzyme-linked immunosorbent assay (ELISA) kits purchased from Shanghai Enzyme-linked Biotechnology Co., Ltd. (Shanghai, China). The catalog numbers were as follows: E2 (ml038322), BALP (ml003415), BGP (ml106924), TRAP (ml106987), and PINP (ml105054). All assays were performed strictly in accordance with the manufacturer’s instructions.

### 2.7. Hematoxylin-Eosin (HE) Staining

Femoral tissues were decalcified, paraffin-embedded, and sectioned at 4 µm for hematoxylin-eosin (HE) staining.

### 2.8. Oil Red O Staining

Following fixation and paraffin embedding, liver sections (4 µm) were stained with Oil Red O, counterstained with Mayer’s hematoxylin, and coverslipped. Quantitative analysis was performed using ImageJ 1.54p software.

### 2.9. Tissue TSA Staining

Deparaffinized sections underwent antigen retrieval, followed by the sequential application of primary antibodies, secondary antibodies, and TSA fluorophores. The staining cycle was repeated after antibody elution. Sections were then counterstained with DAPI and mounted in anti-fade medium, with images captured using a multi-channel fluorescence slide scanner.

### 2.10. Cell Immunofluorescence Staining

Following fixation and blocking, cells were incubated with primary and secondary antibodies, counterstained with DAPI, and examined under a confocal laser scanning microscope (Zeiss, Oberkochen, Germany).

### 2.11. Alkaline Phosphatase Staining (ALP)

BMSCs were treated for 5 days, fixed, and stained for ALP using the Alkaline Phosphatase Color Development Kit (Beyotime Biotechnology, Shanghai, China; catalog No. C3250S) according to the manufacturer’s instructions. Images were captured under an inverted microscope, and ALP staining was semi-quantified using ImageJ 1.54p software (positive area or optical density).

### 2.12. Alizarin Red S Staining (ARS)

BMSCs were osteogenically induced with drugs (medium refreshed every 2–3 days), fixed at day 21/28, and stained with Alizarin Red S. Images were taken under an inverted microscope (Olympus Corporation, Hachioji, Tokyo, Japan). For quantification, stained nodules were dissolved in 10% cetylpyridinium chloride and read on a plate reader (BioTek Epoch; Agilent Technologies, Santa Clara, CA, USA).

### 2.13. mCherry-GFP-LC3 Dual-Fluorescence Autophagic Flux Assay

mCherry-GFP-LC3-labeled BMSCs were seeded in confocal dishes, treated, fixed, and mounted. Images were captured under confocal microscopy (Zeiss, Oberkochen, Germany; 488/561 nm).

### 2.14. Extraction of Nuclear and Cytoplasmic Proteins

Nuclear and cytoplasmic proteins were extracted from BMSCs using the Nuclear and Cytoplasmic Protein Extraction Kit (Beyotime Biotechnology, Shanghai, China; catalog No. P0028) according to the manufacturer’s instructions. All protein samples were kept on ice throughout the procedure. Protein concentrations were determined using the BCA Protein Assay Kit (Beyotime Biotechnology, Shanghai, China; catalog No. P0010S), and 20–30 μg of protein per lane was loaded for Western blot analysis. Membranes were blocked with 5% BSA in TBST. After incubation with primary and secondary antibodies, protein bands were visualized using ECL chemiluminescence substrate (Epizyme Biomedical Technology Co., Ltd., Shanghai, China; catalog No. SQ201) and captured using a chemiluminescence imaging system. Tubulin and histone H3 were used as loading controls for cytoplasmic and nuclear fractions, respectively.

### 2.15. Construction of LV-shTFEB Lentivirus and Generation of Stable Knockdown Cell Lines

LV-shTFEB lentivirus carrying shRNA against rat TFEB (Tfeb-Rat-364, target sequence: CAGGCCGTCATGCATTACATG) and scramble control were obtained from Gene Pharma. BMSCs were infected at MOI 100, selected with 2 μg/mL puromycin, and TFEB silencing was confirmed via Western blot analysis.

### 2.16. Cellular Thermal Shift Assay (CETSA)

BMSCs treated with nuciferine (1 μmol/L) or DMSO were heated at 44–64 °C for 3 min, frozen at −80 °C overnight, and subjected to three liquid nitrogen freeze–thaw cycles (5 min each). Lysates were cleared by centrifugation (4 °C, 12,000× *g*, 25 min), and supernatants were immunoblotted.

### 2.17. Western Blot Analysis

Cells were lysed in RIPA, cleared by centrifugation, and quantified by BCA. Equal protein loads were separated via SDS-PAGE, transferred, blocked, and immunoblotted. Bands were developed using ECL reagents, imaged, and quantified via densitometric analysis. The original Western blot images can be found in the Supplementary Materials.

### 2.18. RT-qPCR

Total RNA was extracted from BMSCs using TRIzol reagent (Invitrogen, Carlsbad, CA, USA;AM9738). cDNA was synthesized using the Evo M-MLV Reverse Transcription Kit (Accurate Biology, Changsha, China; AG11728). RT-qPCR was performed using the SYBR Green Pro Taq HS Premix Kit (Accurate Biology, AG11701). The thermal cycling protocol was as follows: initial denaturation at 95 °C for 30 s, followed by 40 cycles of 95 °C for 5 s and 60 °C for 30 s. Each sample was run in triplicate. GAPDH served as the internal control. Relative mRNA expression was calculated using the 2^−ΔΔCt^ method. Primer sequences are listed in Supplementary Table S.2.

### 2.19. Statistical Analysis

Sample size was determined based on previous literature and our institutional experience. A post hoc power analysis based on the primary outcome measure indicated that the sample size provided adequate power to detect the observed differences. Data are expressed as the mean ± SD. Intergroup comparisons were performed via analysis of variance. For data meeting the homogeneity of variance assumption, one-way ANOVA was used, followed by Tukey’s HSD test for post hoc pairwise comparisons when *p* < 0.05. For data with unequal variances, the Brown–Forsythe and Welch tests were used, followed by Dunnett’s T3 test for post-hoc pairwise comparisons when *p* < 0.05. A *p*-value < 0.05 was considered statistically significant.

## 3. Results

### 3.1. Nue Promotes Bone Formation in Ovariectomized Rats

**Figure 1:**
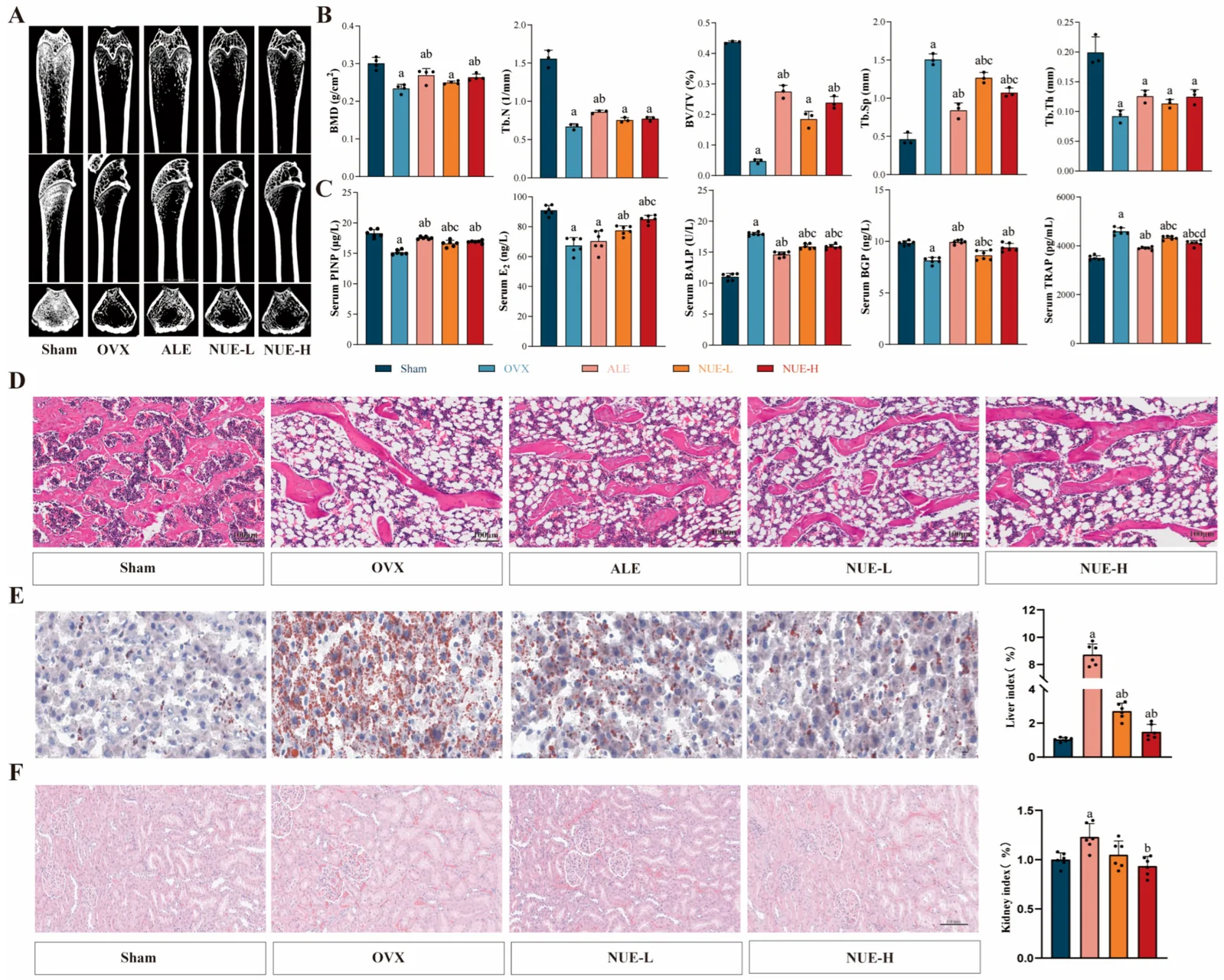
Effects of Nue on bone microarchitecture, serum biomarkers, and tissue histology in OVX rats. (**A**) Two-dimensional micro-CT tomographic images; (**B**) BMD and bone microstructural parameters BV/TV, Tb.N, Tb.Sp, and Tb.Th of the femur (*n* = 4); (**C**) Serum E2, BALP, PINP, BGP, and TRAP measured via ELISA (*n* = 6); (**D**) Femoral pathological morphology observed via HE staining (*n* = 6, Scale bar = 100 µm); (**E**) Oil Red O staining of liver tissue (*n* = 6, Scale bar = 20 µm); (**F**) HE staining of kidney tissue (*n* = 6, Scale bar = 100 µm). Data are expressed as mean ± SD, ^a^ *p* < 0.05 vs. Sham, ^b^ *p* < 0.05 vs. OVX, ^c^ *p* < 0.05 vs. ALE, ^d^ *p* < 0.05 vs. Nue-L.

Micro-CT images of the distal femoral sections can be seen in Figure 1A. In sham-operated rats, the femoral trabeculae were dense, regularly arranged and continuous, whereas in the OVX group, the trabeculae were markedly reduced, thinned, and fractured, with an enlarged marrow cavity. In comparison to the OVX group, the ALE, Nue-L, and Nue-H groups all exhibited improved trabecular structures to varying degrees, manifested as increased trabecular number and better structural continuity. Bone mineral density (BMD) and trabecular bone parameter analysis revealed (Figure 1B) that, relative to the Sham group, the OVX group displayed marked deterioration in BMD and trabecular microarchitecture (reduced BV/TV, Tb. Th, and Tb. N; elevated Tb. Sp). After Nue intervention, these microstructural impairments were markedly ameliorated. The improvement in the Nue-H group was more pronounced than that in the Nue-L group and showed a trend similar to the ALE group. The data suggest that Nue can attenuate estrogen deficiency-induced trabecular loss and microstructural damage.

In addition, ELISA measurement of serum E2 and bone metabolism-related indicators showed (Figure 1C) that serum E2 levels dropped markedly in OVX rats versus Sham controls, which verified that the ovariectomy model was effectively established. Nue treatment upregulated the serum levels of E2, BGP, and PINP while reducing the level of the bone resorption marker TRAP. The BALP level was lower in the Nue group than in the OVX group, but still higher than that in the sham group. These results suggest that Nue may ameliorate the abnormal bone metabolism of OVX rats from two aspects, namely promoting osteogenic activity and inhibiting excessive bone resorption. HE staining corroborated the micro-CT observations and bone parameter analyses (Figure 1D), showing that after Nue intervention, the number and area of trabeculae in OVX rats increased and structural continuity improved, with the Nue-H group showing more obvious recovery of trabecular morphology.

To evaluate the in vivo safety of Nue, we observed hepatic lipid accumulation and renal histopathological changes across all groups via Oil Red O staining and HE staining. Oil Red O staining showed that the hepatic lipid droplet signal was markedly greater in the OVX group than in the Sham group, whereas the positive signal area was markedly reduced after Nue intervention, with neatly arranged hepatic cords and no pathological changes such as steatosis, inflammatory cell infiltration, or necrosis (Figure 1E). HE staining of kidney sections revealed that, following Nue treatment, the glomeruli, tubules, and renal interstitium remained well-defined and intact, with no evident histopathological alterations indicative of toxicity (Figure 1F). These results indicate that, at the dosage and duration used in this experiment, Nue did not cause obvious hepato- or nephrotoxicity and ameliorated OVX-induced hepatic lipid accumulation, suggesting a favorable in vivo safety profile.

### 3.2. Nue Upregulates TFEB Expression and Affects the Expression of Autophagy Pathway Markers in Bone Tissue of OVX Rats

**Figure 2:**
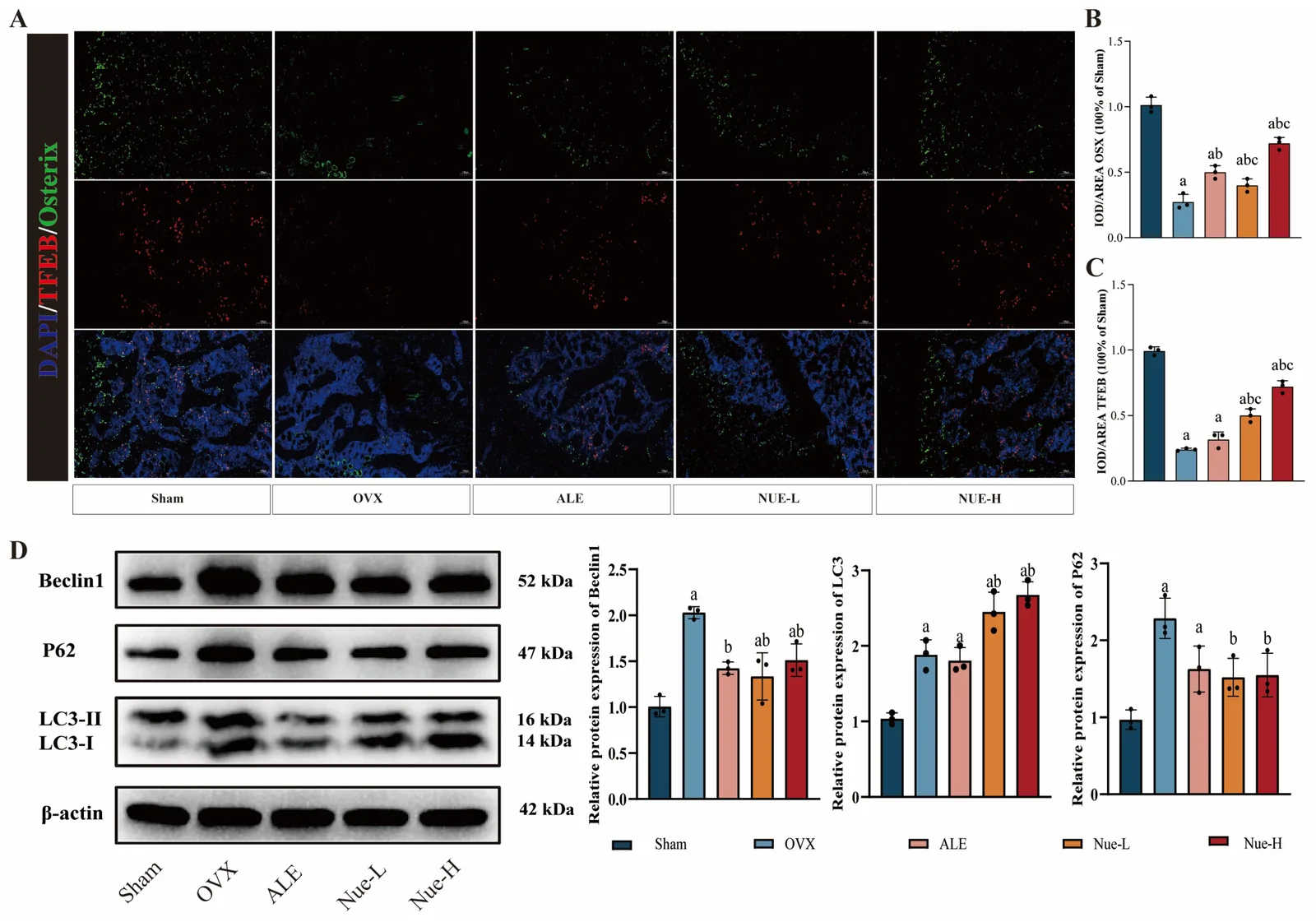
Expression of OSX and key proteins of the TFEB-autophagy pathway in the bone tissue of OVX rats. (**A**) Expression of OSX and TFEB in bone tissue detected via TSA staining. (Scale bar = 100 µm); (**B**) Semi-quantitative analysis of the mean fluorescence intensity of OSX; (**C**) Semi-quantitative analysis of the mean fluorescence intensity of TFEB; (**D**) Protein expression levels of p62, Beclin1, and the LC3-II/LC3-I ratio in bone tissue detected via Western blotting and a relative quantitative analysis (*n* = 3). Data are expressed as mean ± SD, ^a^ *p* < 0.05 vs. Sham, ^b^ *p* < 0.05 vs. OVX, ^c^ *p* < 0.05 vs. ALE. The original WB image can be found in the Supplementary Materials.

To further evaluate whether Nue can regulate bone formation through TFEB, we observed the localization and expression levels of the osteogenesis-related transcription factor OSX and TFEB in femoral bone tissue. The TSA staining results showed (Figure 2A) that OSX expression was detected in the growth plate and trabeculae of Sham group femurs, whereas TFEB was mainly distributed below the growth plate and around the trabeculae, and co-localized expression of the two was observed. These results suggest that TFEB may affect bone formation mainly by participating in osteoblast differentiation and mineralization around the trabeculae. Semi-quantitative analysis showed (Figure 2B,C) that, compared to the Sham group, the expression of OSX and TFEB in the bone tissue of OVX rats was significantly decreased, and the distribution of OSX in the growth plate region was markedly reduced. Compared to the OVX group, ALE treatment upregulated OSX levels in bone tissue but did not markedly alter TFEB expression. Compared to the ALE group, Nue intervention simultaneously increased the expression of OSX and TFEB. This enhancement was more pronounced in the Nue-H group, which exhibited particularly increased OSX expression in the growth plate region, suggesting that Nue intervention may also promote bone formation by affecting endochondral ossification.

We further detected the protein levels of the autophagy markers LC3, P62, and Beclin1 in bone tissue via Western blotting (Figure 2D). Compared to the Sham group, the levels of LC3-II/LC3-I, Beclin1, and p62 were elevated in the OVX group, suggesting that autophagy in OVX rats may be in a blocked state. Following Nue intervention, the LC3-II/LC3-I ratio was further elevated, while p62 expression decreased to varying degrees, and Beclin1 expression decreased compared with the OVX group but remained higher than that in the Sham group, most notably in the Nue-H group. These results suggest that Nue may ameliorate OVX-induced autophagic impairment and restore autophagic degradation function.

### 3.3. Nue Facilitates Osteogenic Differentiation of BMSCs

**Figure 3:**
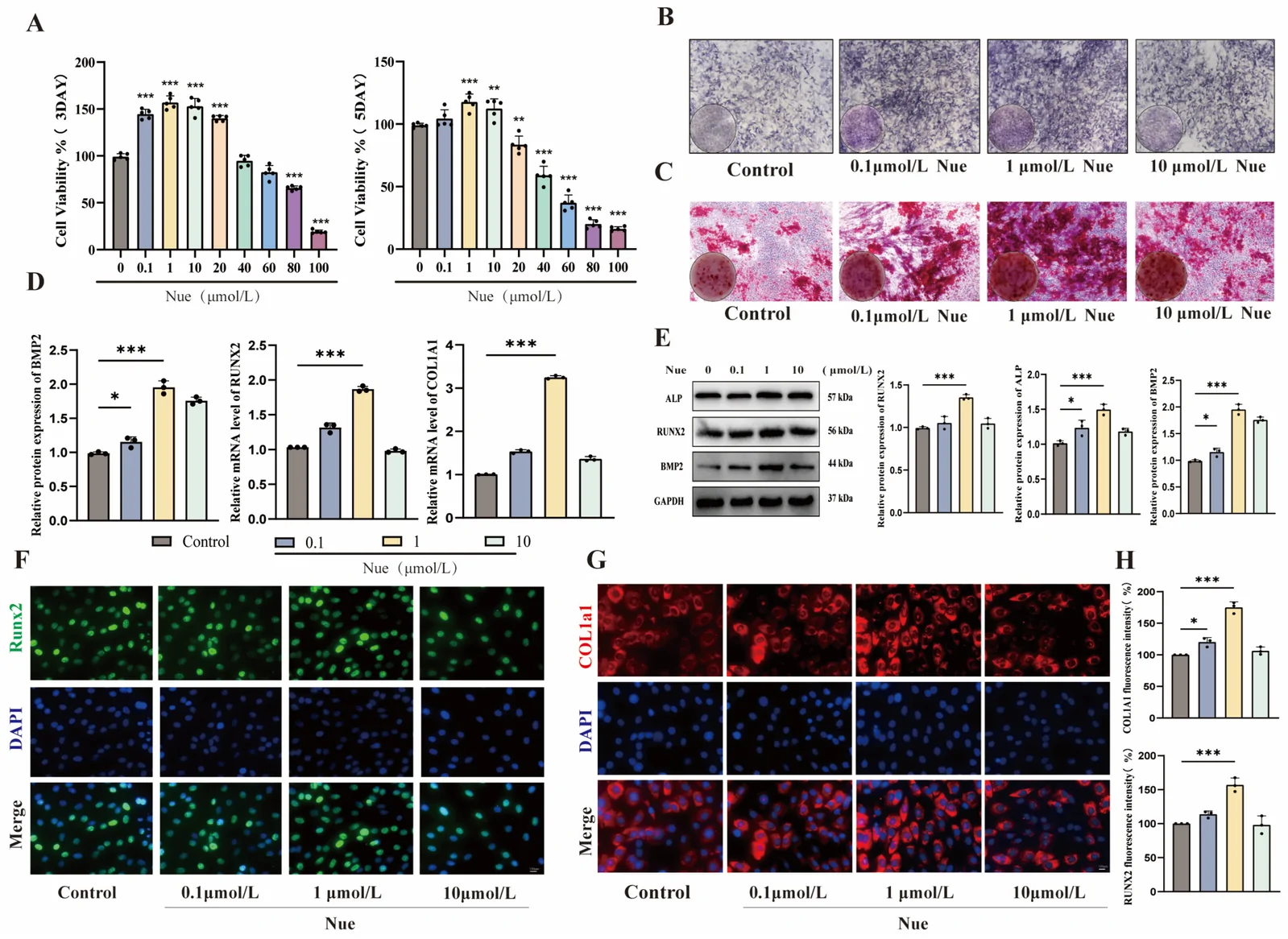
Effects of Nue on the osteogenic differentiation of BMSCs. (**A**) Effects of different concentrations of Nue on the viability of BMSCs detected by the CCK-8 assay; (**B**) ALP staining (scale bar = 200 µm); (**C**) ARS staining (scale bar = 200 µm); (**D**) mRNA expression levels of the osteogenesis-related genes COL1A1, RUNX2, and BMP2 in BMSCs after Nue treatment detected by RT-qPCR; (**E**) Protein expression levels of ALP, RUNX2, and BMP2 in BMSCs after Nue treatment detected via Western blotting and relative quantitative analysis; (**F**,**G**) Immunofluorescence staining showing the expression and localization of RUNX2 (F) and COL1A1 (G) in BMSCs after Nue treatment (Scale bar = 100 µm); (**H**) Quantitative analysis of the relative fluorescence intensity of RUNX2 and COL1A1 in (**F**,**G**). Data are expressed as mean ± SD, * *p* < 0.05, ** *p* < 0.01, *** *p* < 0.001. The original WB image can be found in the Supplementary Materials.

To clarify how Nue regulates the osteogenic differentiation of BMSCs, we first assessed Nue’s impact on BMSC viability via the CCK-8 assay (Figure 3A). After treatment of BMSCs for 3 or 5 days, Nue in the concentration range of 0.1–10 µmol/L had no significant effect on BMSC viability. Nue at concentrations of 0.1, 1, and 10 µmol/L was further selected as the intervention conditions. After 7 days of treatment, alkaline phosphatase (ALP) staining (Figure 3B) showed that alkaline phosphatase activity was the strongest in the 1 µmol/L group. After 28 days of osteogenic induction, Alizarin Red S (ARS) staining (Figure 3C) revealed that mineralized nodule formation in all Nue groups was markedly increased, with 1 µmol/L Nue showing the most significant effect.

The RT-qPCR results showed (Figure 3D) that Nue (0.1, 1, and 10 µmol/L) treatment markedly upregulated the mRNA levels of the osteogenesis-related factors COL1A1, BMP2, and RUNX2. The Western blot results showed that Nue (0.1, 1, and 10 µmol/L) also markedly promoted the protein levels of RUNX2, ALP, and BMP2, with the most significant upregulation in the 1 µmol/L group, consistent with the trend in the RT-qPCR results (Figure 3E). The immunofluorescence staining results (Figure 3F,G) further showed that 1 µmol/L Nue treatment significantly enhanced the fluorescence signal intensity of RUNX2 and COL1A1. These data confirm that Nue facilitates the osteogenic differentiation of BMSCs, and based on these experiments, 1 µmol/L Nue was used as the optimal intervention concentration in subsequent mechanistic studies.

### 3.4. Nue Upregulates TFEB Expression and Induces Its Nuclear Translocation to Promote the Osteogenic Differentiation of BMSCs

**Figure 4:**
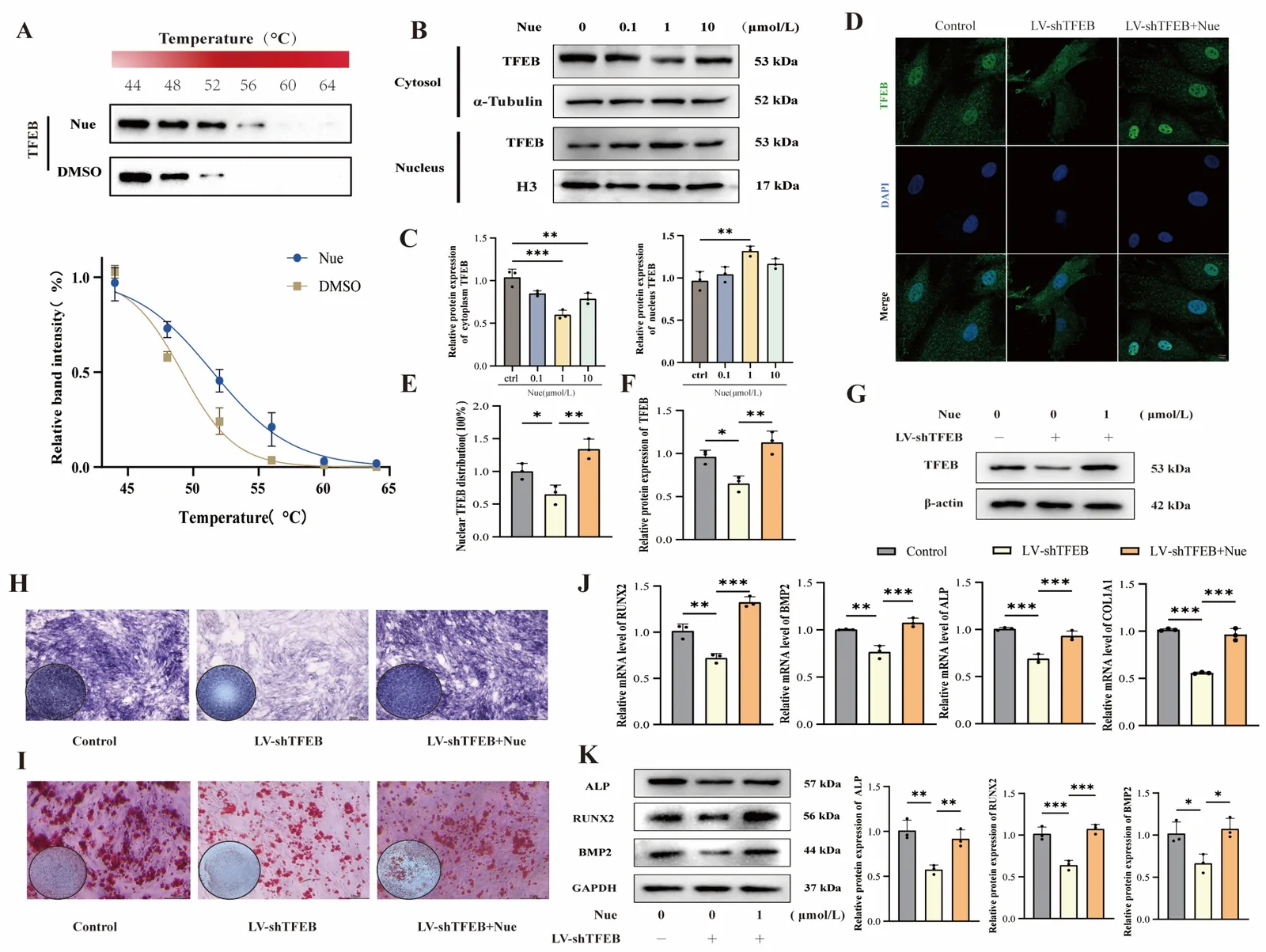
Nue promotes osteogenic differentiation by upregulating TFEB expression and inducing its nuclear translocation. (**A**) CETSA analysis of TFEB protein stability upon Nue treatment; (**B**) Effect of Nue on TFEB protein expression in the cytoplasm and nucleus of BMSCs detected via Western blotting; (**C**) Relative quantification of the proteins shown in (**B**). (**D**) Localization and expression of TFEB in the control group, LV-shTFEB group, and LV-shTFEB+Nue group observed via immunofluorescence staining (scale bar = 20 µm); (**E**) Relative quantification of TFEB fluorescence intensity shown in (**D**); (**F**) Relative quantification of the proteins shown in (**G**); (**G**) TFEB protein expression levels in each group detected via Western blotting; (**H**) ALP staining (scale bar = 200 µm); (**I**) ARS staining (scale bar = 200 µm); (**J**) mRNA expression levels of the osteogenesis-related genes COL1A1, BMP2, ALP, and RUNX2 in each group detected via RT-qPCR and relative quantitative analysis; (**K**) Protein expression levels of BMP2, ALP, and RUNX2 in each group detected via Western blotting and relative quantitative analysis. Data are expressed as mean ± SD, * *p* < 0.05, ** *p* < 0.01, *** *p* < 0.001. The original WB image can be found in the Supplementary Materials.

To explore how Nue activates TFEB, we first assessed the effect of Nue on TFEB protein stability. CETSA revealed that, in the DMSO group, TFEB protein levels progressively declined with increasing temperature (44 °C to 64 °C) (Figure 4A). By contrast, in the Nue-treated group (1 µmol/L), the remaining amount of TFEB protein at multiple temperature points (especially in the range of 52~64 °C) was markedly elevated compared with that in the control group, indicating that Nue enhances the thermal stability of TFEB protein in BMSCs.

To verify the effect of Nue on TFEB expression, we first detected the effect of Nue intervention on the levels of TFEB in the nucleus and cytoplasm of BMSCs. The Western blot results showed (Figure 4B) that Nue treatment increased TFEB protein expression in the nucleus, accompanied by a corresponding decrease in TFEB expression in the cytoplasm, indicating that Nue can induce the nuclear translocation of TFEB. The accumulation of nuclear TFEB was most significant in the 1 µmol/L group, consistent with the aforementioned optimal concentration.

We constructed LV-shTFEB, and Western blot analysis verified (Figure 4F,G) that the total TFEB level in the LV-shTFEB group was markedly reduced relative to the control group, confirming knockdown efficiency. By contrast, TFEB protein abundance in the LV-shTFEB+Nue group was significantly restored relative to that in the LV-shTFEB group alone, indicating that Nue can partially restore TFEB expression. The immunofluorescence staining results showed (Figure 4D,E) that the TFEB level was significantly decreased in the LV-shTFEB group, while the LV-shTFEB+Nue group not only showed a significant recovery of total fluorescence intensity compared to the LV-shTFEB group alone but also showed a tendency toward nuclear enrichment of the fluorescence signal. These results indicate that Nue can partially restore TFEB expression and maintain its nuclear translocation ability even under the knockdown background.

Building on these findings, to further clarify the necessity of TFEB in the osteogenic action of Nue, we observed the effects of LV-shTFEB and LV-shTFEB+Nue on BMSC osteogenesis. ALP and ARS staining revealed (Figure 4H,I) that ALP activity was notably diminished and the formation of mineralized nodules was markedly decreased in the LV-shTFEB group. By contrast, the ALP activity and mineralized area in the LV-shTFEB+Nue group were notably restored relative to the LV-shTFEB group alone, suggesting that Nue can partially rescue the LV-shTFEB-induced inhibition of osteogenic differentiation. RT-qPCR analysis revealed (Figure 4J) that relative to the LV-shTFEB group, the LV-shTFEB+Nue group significantly restored the mRNA levels of the osteogenic markers COL1A1, ALP, BMP2, and RUNX2. Western blot data confirmed (Figure 4K) that Nue also notably restored the expression of RUNX2, ALP, and BMP2 inhibited by LV-shTFEB. In summary, Nue can promote the upregulation and nuclear translocation of TFEB, LV-shTFEB can inhibit BMSC osteogenic differentiation, and Nue intervention can partially reverse this inhibition, indicating that Nue promotes the osteogenic differentiation of BMSCs by elevating TFEB levels and inducing its translocation to the nucleus.

### 3.5. Nue Promotes the Osteogenic Differentiation of BMSCs by Activating the TFEB-Mediated Autophagy-Lysosomal Pathway

**Figure 5:**
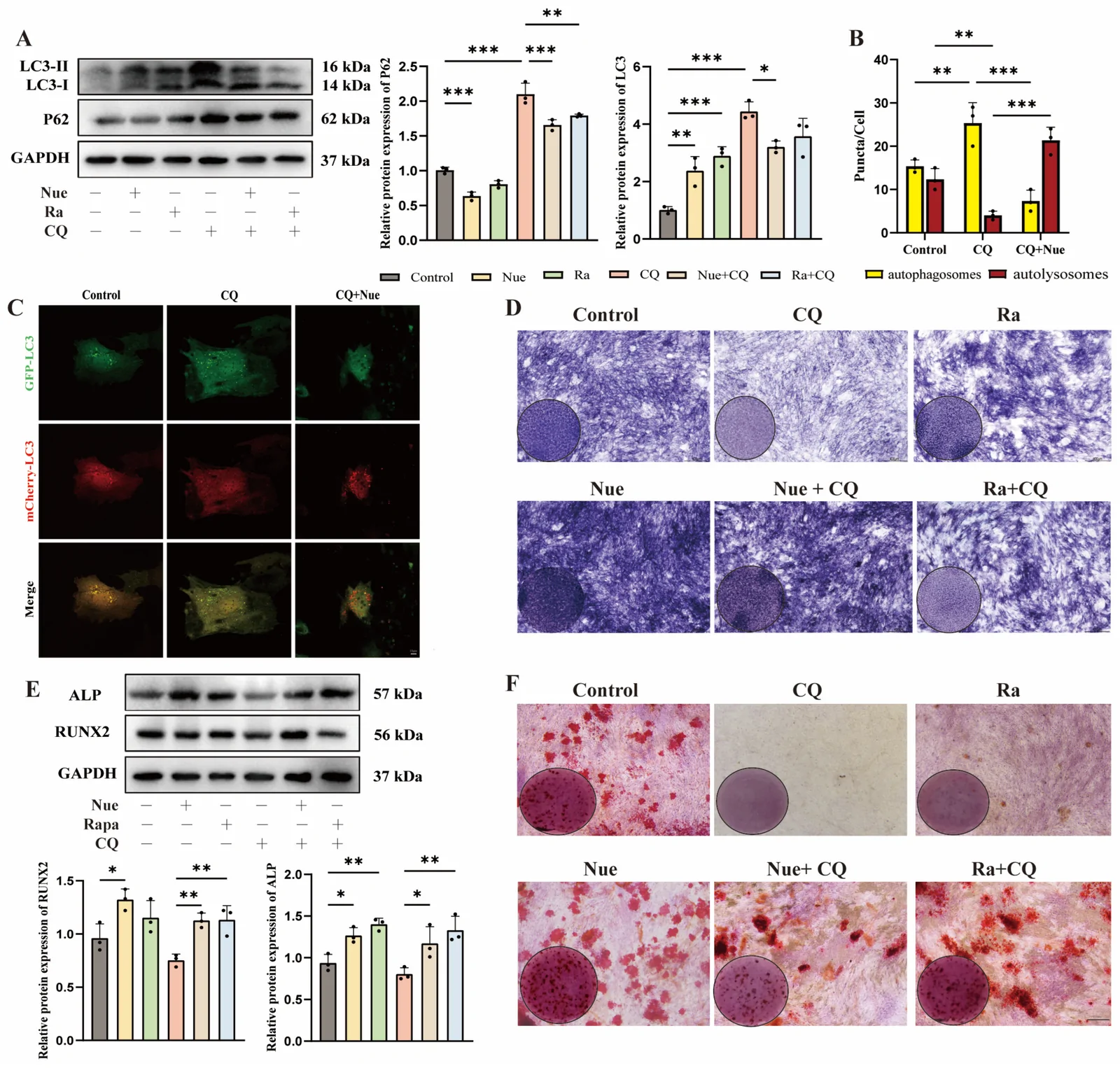
Nue activates the TFEB-mediated autophagy-lysosomal pathway to promote osteogenic differentiation. (**A**) Protein expression levels of p62 and the LC3-II/LC3-I ratio in each group detected via Western blotting and relative quantitative analysis; (**B**) Quantification of EGFP and mCherry positive fluorescent puncta per individual cell in (**C**); (**C**) Changes in autophagic flux in each group detected using the mCherry-GFP-LC3 dual-fluorescence autophagy indicator system (scale bar = 10 µm); (**D**) ALP staining of each group (scale bar = 400 µm); (**E**) Protein expression levels of RUNX2 and ALP detected via Western blotting and relative quantitative analysis; (**F**) ARS staining of each group (scale bar = 200 µm); Data are expressed as mean ± SD. * *p* < 0.05, ** *p* < 0.01, *** *p* < 0.001. The original Western blot images can be found in the Supplementary Materials.

To determine whether Nue facilitates osteogenic differentiation through the autophagy pathway, we detected changes in autophagy marker proteins via Western blotting. The results are presented in Figure 5A. Relative to the blank group, the LC3-II/LC3-I ratio in the Nue-treated group was markedly elevated, whereas the levels of the autophagy substrate p62 dropped notably. The rapamycin (Rapa, autophagy activator) treatment group exhibited a similar pattern to the Nue group, whereas the chloroquine (CQ, autophagy-lysosomal inhibitor) treatment group displayed an elevated LC3-II/LC3-I ratio accompanied by massive accumulation of p62, indicating that autophagic flux may be blocked. Compared to the CQ group, LC3-II further accumulated in the CQ+Nue group, suggesting that Nue may promote autophagosome synthesis. The CQ+Ra group also showed a similar trend, further confirming the consistency of the action mode between Nue and the known autophagy activator, jointly supporting that Nue may enhance autophagic flux by promoting autophagosome synthesis. The mCherry-GFP-LC3 tandem fluorescent reporter (Figure 5B,C) was used to assess autophagic flux. According to the results, a small number of scattered yellow puncta and red puncta were observed in the blank group, reflecting the normal operation of basal autophagic flux. The CQ-treated group showed massive accumulation of yellow puncta with almost complete disappearance of red puncta, indicating that the autophagic degradation process is effectively blocked. Compared to the CQ group alone, red puncta reappeared in the CQ+Nue co-treatment group, and the accumulation of yellow puncta was markedly alleviated, suggesting that Nue can partially restore the autophagic flux inhibited by CQ.

Based on these results, we further verified through functional experiments whether Nue promotes osteogenic differentiation through the TFEB-mediated autophagy-lysosomal pathway. The ALP staining results showed (Figure 5D) that ALP activity in the Nue and Ra groups was markedly enhanced relative to the control group, whereas ALP staining intensity in the CQ group was markedly weakened, indicating that blocking autophagic flux can significantly inhibit early osteogenic differentiation. ALP activity in the CQ+Nue group was partially restored relative to the CQ group, and the CQ+Ra group also revealed a similar improving tendency. The ARS staining findings (Figure 5F) aligned with the trend in ALP staining. Western blot detection of the levels of osteogenic markers showed (Figure 5E) that RUNX2 and ALP protein levels in the Nue and Ra groups were significantly upregulated compared to the control group, whereas the levels of the above proteins were markedly suppressed in the CQ group. The protein levels of RUNX2 and ALP in the CQ+Nue group were restored to some extent compared to the CQ group, and the CQ+Ra group exhibited a similar recovery pattern. These findings suggest that Nue can facilitate BMSC osteogenesis through activation of the TFEB-mediated autophagy-lysosomal pathway.

## 4. Discussion

The core pathological basis of postmenopausal osteoporosis is a bone remodeling imbalance caused by estrogen deficiency. Specifically, enhanced bone resorption with relatively insufficient osteogenesis eventually causes decreased bone mass, destruction of trabecular microarchitecture, and reduced bone strength [23]. An OVX-induced osteoporosis model was employed to mimic the postmenopausal osteoporotic state, and the interventional effect of Nue on bone formation was evaluated by focusing on bone microarchitecture, serum bone turnover markers, and histomorphology. Micro-CT and bone parameter analysis are important methods for evaluating changes in trabecular microarchitecture and can intuitively reflect alterations in bone mass and the spatial structure of bone tissue. In this study, the distal femoral trabeculae of OVX rats were sparse, thinned, and fractured, and BMD alterations and related indicators were consistent with the pathological characteristics of postmenopausal osteoporosis. After Nue intervention, the above bone microstructural parameters were improved to varying degrees, and the high-dose Nue group in particular showed more obvious trabecular preservation and structural recovery. Serum bone turnover markers further supported the influence of Nue on the balance of bone remodeling. After OVX, rat bone metabolism is in a high-turnover state, usually manifested as elevated bone resorption-related indicators, while the bone formation process is inhibited or insufficient to compensate for excessive bone resorption [24]. This study showed that Nue can simultaneously enhance osteogenic activity and inhibit osteoclast-mediated bone matrix degradation. OSX is an essential osteogenic transcription factor and is critical for the differentiation of pre-osteoblasts into mature osteoblasts [25]. This study also showed that after Nue treatment, the OSX level in the growth plate and bone marrow cavity of OVX rat bone tissue was increased, with the high-dose Nue group showing more obvious enhancement. These findings suggest that Nue plays a bidirectional modulatory role in the improvement of osteoporosis.

TFEB is an important transcription factor regulating lysosomal biogenesis and autophagic function and has been increasingly linked to bone metabolic homeostasis in recent years [26]. Osteoblasts need to maintain good intracellular protein turnover, organelle quality control, and energy metabolism during differentiation, matrix synthesis, and mineralization, and the TFEB-mediated autophagy-lysosomal pathway is one of the important mechanisms maintaining these functions [27,28]. Under OVX conditions, oxidative stress, inflammatory responses, and cellular metabolic disorders can impair osteoblast function, thereby affecting bone formation. We found that Nue simultaneously enhanced the levels of TFEB and OSX in bone tissue, suggesting that it may, on the one hand, enhance the functional homeostasis of osteoblasts and, on the other hand, promote the initiation of the osteogenic differentiation program, thereby jointly promoting bone formation. In addition, the high-dose Nue group showed a more obvious improving trend than the low-dose group across multiple indicators, including bone microstructural parameters, bone turnover markers, and the fluorescence expression levels of TFEB and OSX, suggesting that the interventional effect of Nue on OVX bone loss may be dose-dependent. Moreover, compared to the positive drug alendronate, Nue showed advantages in the expression of some osteogenesis-related indicators, indicating that, beyond the previously reported inhibition of bone resorption, its promotion of bone formation is also significant.

Bone homeostasis relies on an equilibrium between bone resorption and bone formation [29]. As important precursor cells of osteoblasts, BMSCs are a key cell source for maintaining bone formation and bone regeneration capacity [30]. In postmenopausal osteoporosis, the osteogenic-adipogenic differentiation balance of BMSCs is disrupted, with decreased osteogenic capacity and enhanced adipogenic differentiation, leading to insufficient bone formation and aggravated bone marrow adiposity [31,32]. Therefore, restoring BMSC osteogenic potential is considered a key approach for ameliorating osteoporosis [33]. The results of the cell experiments in this study showed that, within the concentration range of 1–10 µmol/L, Nue significantly enhanced BMSC osteogenesis, with increased ALP staining, increased formation of mineralized nodules, and elevated levels of osteogenic markers including RUNX2, ALP, and BMP2. Among them, RUNX2 serves as an essential transcription factor during the early stage of osteogenic differentiation [34], and BMP2 acts as a crucial upstream regulator of osteogenic induction [35]. The simultaneous improvement in the above indicators suggests that the pro-osteogenic effect of Nue is not limited to a certain terminal link but is more likely to run through multiple stages of osteogenic differentiation, providing an experimental basis for in-depth exploration of its pro-osteogenic pharmacological mechanism.

TFEB has attracted much attention because of its core regulatory role in autophagy and lysosomal biogenesis [36]. As part of the MiT/TFE family, TFEB activity is strictly regulated by subcellular localization. It is usually in a relatively inhibited state in the cytoplasm, but once translocated into the nucleus, it can activate the transcription of multiple autophagy- and lysosome-related genes by recognizing CLEAR elements, coordinating intracellular degradation, recycling and metabolic adaptation processes [37]. To date, the role of TFEB in neurodegenerative diseases, metabolic diseases, and tumors has been widely reported [38,39], and current research indicates that TFEB may play a role in the regulation of osteoblast function, sustain bone tissue homeostasis, and facilitate the adaptation of bone-related cells to metabolic stress [40], but the specific mechanism remains insufficiently studied. This study found that Nue can markedly elevate the levels of TFEB in BMSCs and facilitate its nuclear translocation. This finding suggests that the regulation of bone metabolism by Nue does not stay at the level of osteogenic marker expression. Instead, it also activates TFEB, a key transcriptional hub, to remodel the intracellular metabolic regulatory network, thereby improving the impaired osteogenic microenvironment under OVX conditions. Notably, the CETSA results showed that Nue can enhance the thermal stability of TFEB protein in BMSCs, suggesting that Nue may specifically bind to TFEB protein. This discovery helps elucidate the upstream trigger mechanism of Nue-induced TFEB nuclear translocation.

Increased TFEB expression and enhanced nuclear translocation are usually accompanied by activation of the autophagy-lysosomal pathway [41]. Autophagy serves as a critical cellular process for eukaryotic cells to maintain homeostasis, ensuring cell survival and functional homeostasis by clearing damaged proteins and organelles, recycling nutrient substrates, and buffering stress injury [42,43]. For BMSCs, moderate autophagy helps maintain their activity and promotes osteogenic differentiation, whereas impaired autophagy can lead to the accelerated senescence of BMSCs, mitochondrial dysfunction, and decreased osteogenic capacity, eventually aggravating bone loss [44]. In this study, after Nue treatment, the LC3-II/LC3-I ratio increased and p62 expression decreased, and this trend was consistent with the characteristic actions of the classic autophagy activator rapamycin, indicating that Nue can regulate the autophagic process of BMSCs. Given that TFEB is the core upstream factor initiating the transcription of autophagy-lysosomal genes, the concomitant enhancement of TFEB nuclear translocation induced by Nue suggests that it may drive this autophagic process by activating TFEB transcriptional activity, rather than merely affecting the downstream degradation link of the autophagy pathway [45].

The key function of the autophagy-lysosomal pathway in bone formation may lie in its simultaneous undertaking of multiple functions, such as energy support, organelle quality control, and stress buffering [46]. Autophagy can not only provide metabolic substrates by degrading and recycling intracellular components to meet the continuously increasing biosynthetic demands during differentiation but also reduce the accumulation of reactive oxygen species by clearing damaged mitochondria, thereby avoiding the inhibition of the osteogenic program by oxidative damage [47]. In addition, autophagy may also be involved in matrix protein processing, vesicle transport, and mineralization-related secretory activities, thereby affecting osteogenic maturation and bone matrix mineralization [48]. Combined with the mCherry-GFP-LC3 results, these findings further support that Nue can promote the formation of autophagosomes and maintain their smooth transition to the degradation stage, rather than simply blocking the clearance pathway. This positive regulation of the whole process of autophagic flux may be an important intracellular basis for Nue to effectively support osteogenic differentiation at the functional level.

This study further verified the necessity of TFEB in the pro-osteogenic effect of Nue by knocking down TFEB. The TFEB blockade experiments showed that after TFEB inhibition, ALP staining of BMSCs was weakened, mineralized nodules were reduced, and osteogenesis-related indicators such as RUNX2 and ALP were significantly decreased. These results directly demonstrate that TFEB critically promotes the osteogenic differentiation of BMSCs, corroborating prior studies reporting the involvement of TFEB in stem cell function regulation and tissue regeneration [49]. Under TFEB-suppressed conditions, Nue was still capable of partially reversing the osteogenic impairment of BMSCs, as shown by the recovery of osteogenesis-related molecule expression, ALP activity, and mineralization capacity. In addition, Nue can upregulate TFEB expression and promote its nuclear translocation, indicating that Nue activates the downstream autophagy-lysosomal pathway by restoring the functional expression of TFEB. This partially offset the inhibition of osteogenic differentiation caused by knockdown, suggesting that TFEB plays a central role in mediating the pro-osteogenic effect of Nue.

Current clinical drugs for osteoporosis mainly include two categories, antiresorptive and bone-forming agents. However, their long-term application still faces problems such as efficacy plateaus, adverse reactions, or indication limitations [50]. Natural products, owing to their abundant sources, diverse structures, and multi-target regulatory advantages, hold promise for the intervention of chronic metabolic bone diseases. Nue has previously been reported mainly for its pharmacological effects of lipid regulation, anti-inflammation, anti-oxidation, and improvement of metabolic disorders [51]. Although it has important application potential in the field of bone metabolism, related research is still relatively limited. This study confirms that Nue can not only ameliorate bone mass loss and bone microstructural damage in OVX rats but also enhance the osteogenic differentiation of BMSCs by promoting TFEB nuclear translocation and activating the autophagy-lysosomal pathway. This finding suggests that it may become an anti-osteoporotic candidate molecule from natural medicinal sources with further development value. While evaluating the anti-osteoporotic efficacy of Nue, this study also observed its effects on major organs. The histological results showed that, at the experimental dosage and duration, Nue reduced OVX-induced hepatic lipid accumulation and did not cause obvious renal histological changes. Combined with previous reports that Nue has a favorable safety profile in various animal models, these results provide a preliminary toxicological reference for the further development of Nue as an anti-osteoporotic candidate molecule.

This study has certain limitations. First, this study was mainly validated based on the OVX model, which closely mimics postmenopausal osteoporosis but cannot fully represent the pathological characteristics of senile, glucocorticoid-induced, or secondary osteoporosis; the effect of Nue in other osteoporosis models still needs further evaluation. Second, we only assessed the impact of Nue on the histomorphology of the liver and kidney during the intervention period; its in vivo pharmacokinetic characteristics, bioavailability, tissue distribution, and long-term administration safety remain unclear, and systematic further research is required to evaluate the viability of clinical translation.

In summary, this study demonstrates that Nue can significantly ameliorate the reduction in BMD and bone microstructural damage in OVX rats and promote the osteogenic differentiation of BMSCs. The activation of the autophagy-lysosomal pathway by Nue through upregulating TFEB expression and inducing its nuclear translocation represents a key mechanism through which Nue mediates its anti-osteoporotic effect. This study expands the pharmacological understanding of Nue in the regulation of bone metabolism and further offers experimental evidence for the development of intervention strategies for postmenopausal osteoporosis targeting the TFEB-mediated autophagy-lysosomal pathway. 

## 5. Conclusions

This study examined the bone-protective efficacy of Nue on postmenopausal osteoporosis and its molecular mechanism. In vivo experiments confirmed that Nue can significantly ameliorate the reduction in BMD and the destruction of trabecular microarchitecture in OVX rats and reverse bone mass loss associated with estrogen deficiency. In vitro experiments showed that Nue promotes the osteogenic differentiation of BMSCs in a concentration-dependent manner; upregulates the expression of osteogenesis-related genes and proteins such as RUNX2, ALP, COL1A1, and BMP2; and enhances ALP activity and mineralized nodule formation. Mechanistic studies further demonstrated that Nue activates the TFEB-mediated autophagy-lysosomal pathway by upregulating TFEB expression and inducing its translocation from the cytoplasm to the nucleus, thereby enhancing autophagic flux in BMSCs and exerting a pro-osteogenic effect. TFEB knockdown experiments and autophagic flux blockade experiments jointly verified the necessity of this pathway in the pro-osteogenic effect of Nue. In conclusion, this study reveals the important role of the TFEB–autophagy–lysosomal axis in the regulation of bone formation, clarifies the pharmacological mechanism by which Nue promotes osteogenic differentiation by activating this pathway, and provides new experimental evidence and a candidate molecule for the prevention and treatment of postmenopausal osteoporosis targeting TFEB-mediated autophagy.

## Data Availability

The original contributions presented in this study are included in the article/Supplementary Materials. Further inquiries can be directed to the corresponding authors.

## Supplementary Materials

The following supporting information can be downloaded at: https://www.mdpi.com/article/10.3390/biom16091370/s1, Table S.1: Antibodies and Reagents; Table S.2: Sequences of the primers. The original Western blot images can be found in the Supplementary Materials.
