# Supplementary material for: Nuciferine Ameliorates Ovariectomy-Induced Bone Loss via TFEB-Mediated Autophagy-Lysosomal Pathway
Source: Biomolecules. 2026 Sep 21;16(9):1370. doi: 10.3390/biom16091370 (PMC13604095; doi:10.3390/biom16091370)

Figure 2D-Beclin1

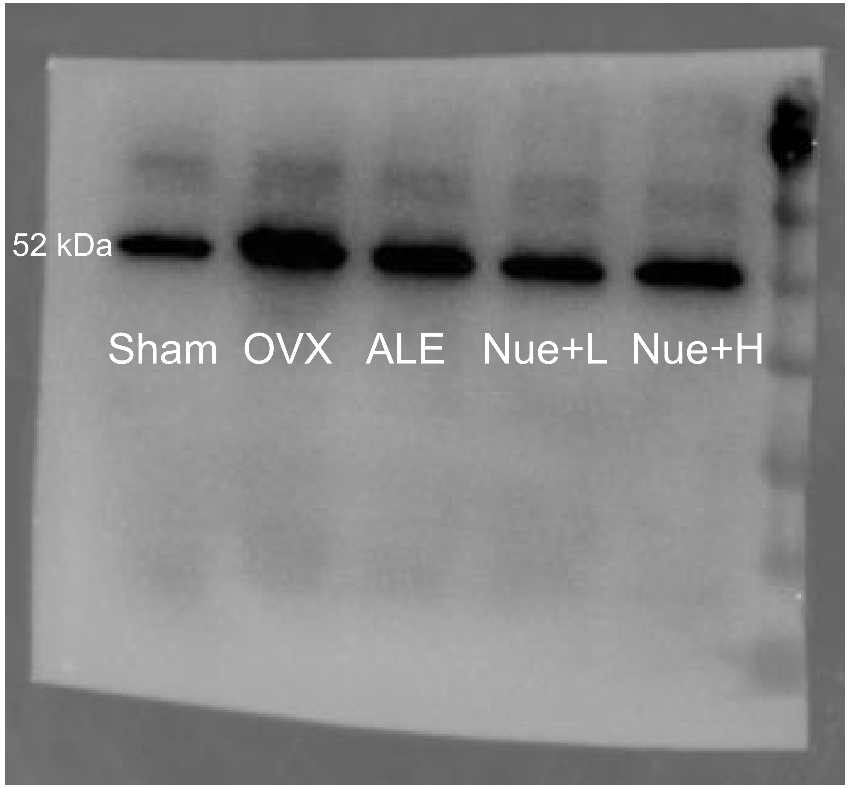

Figure 2D-P62

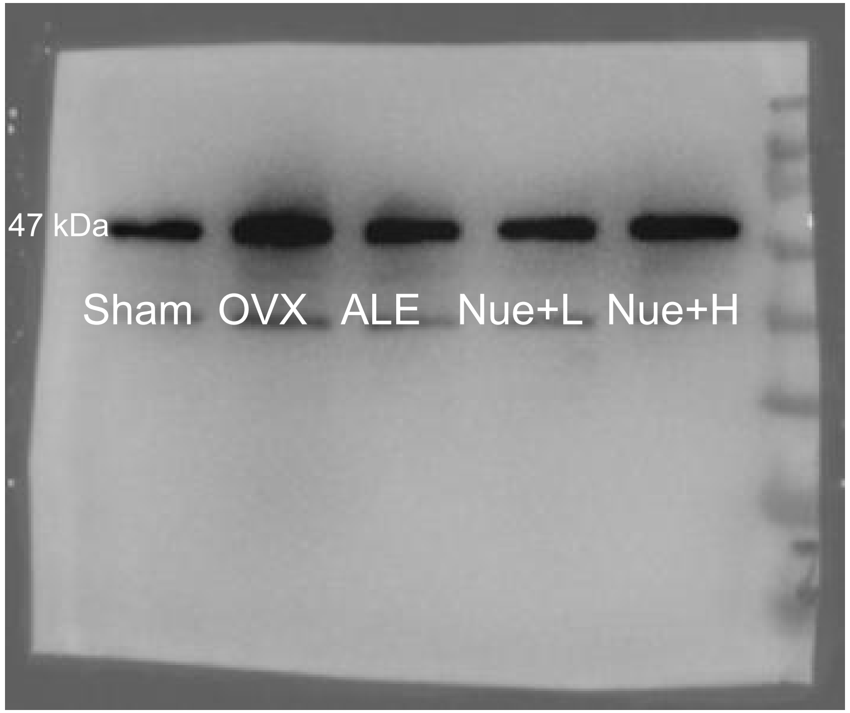

Figure 2D-LC3

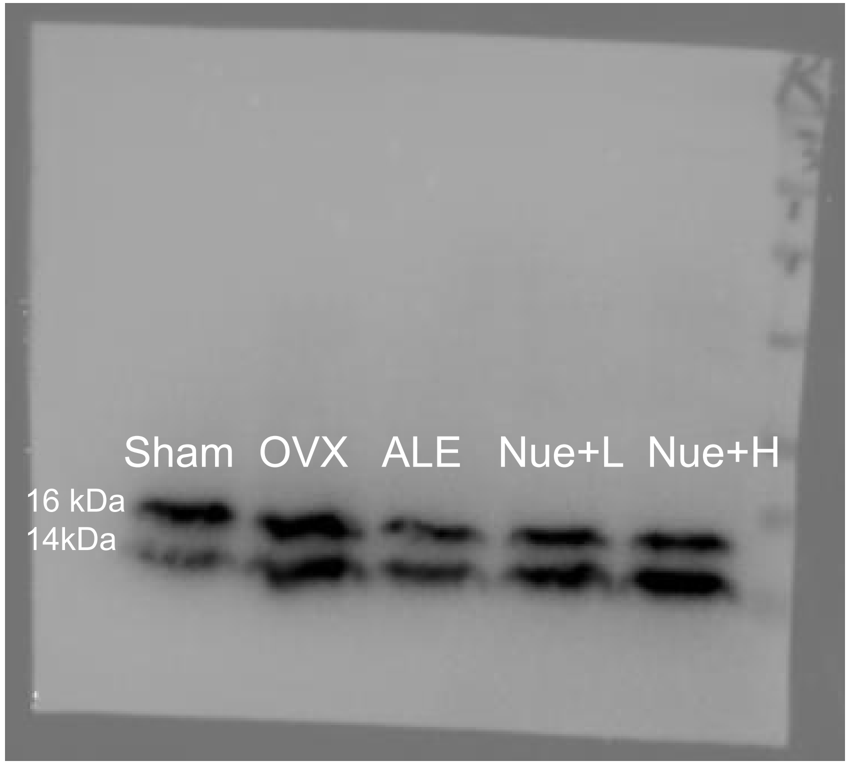

Figure 2D-β-actin

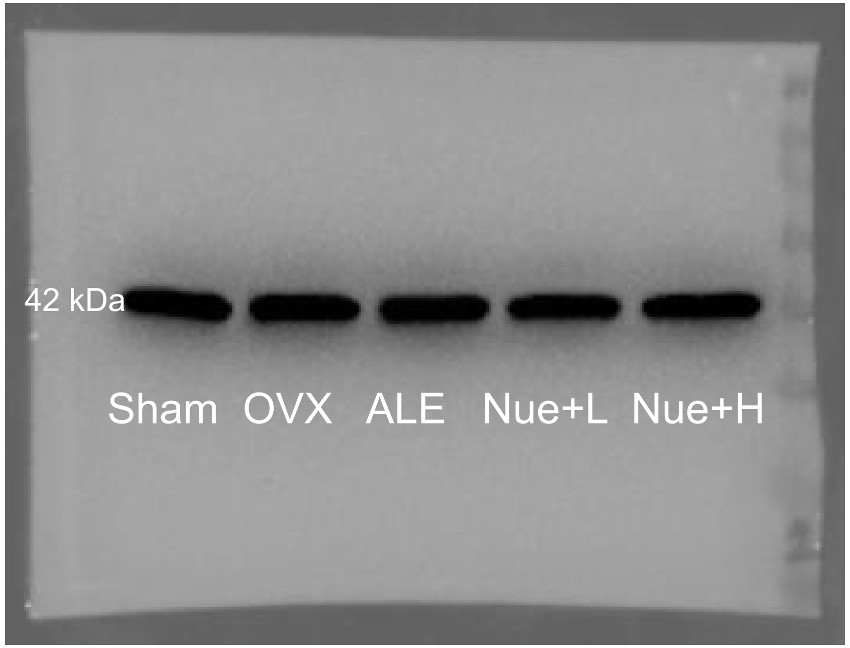

Figure 3E-ALP

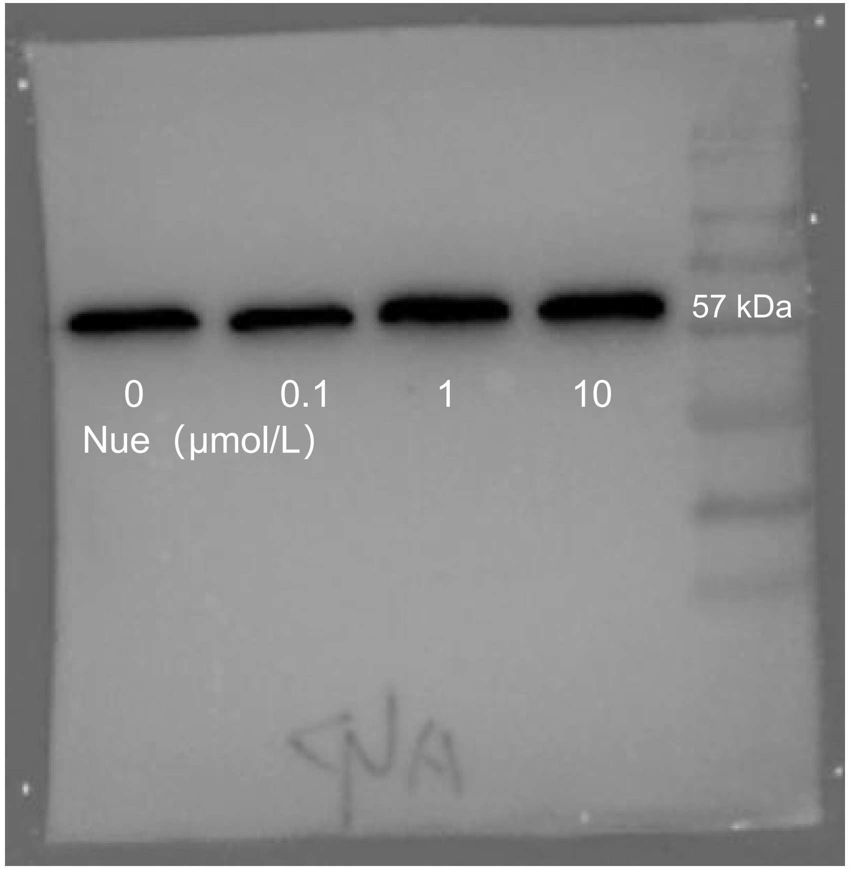

Figure 3E-RUNX2

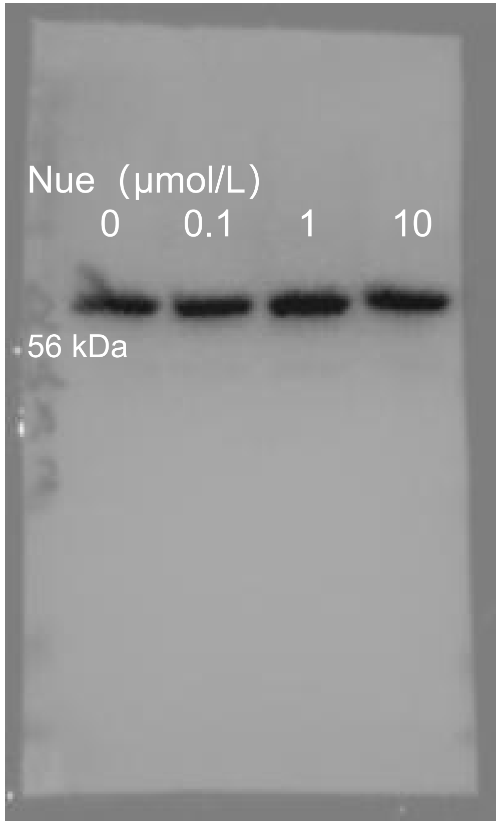

Figure 3E-GAPDH

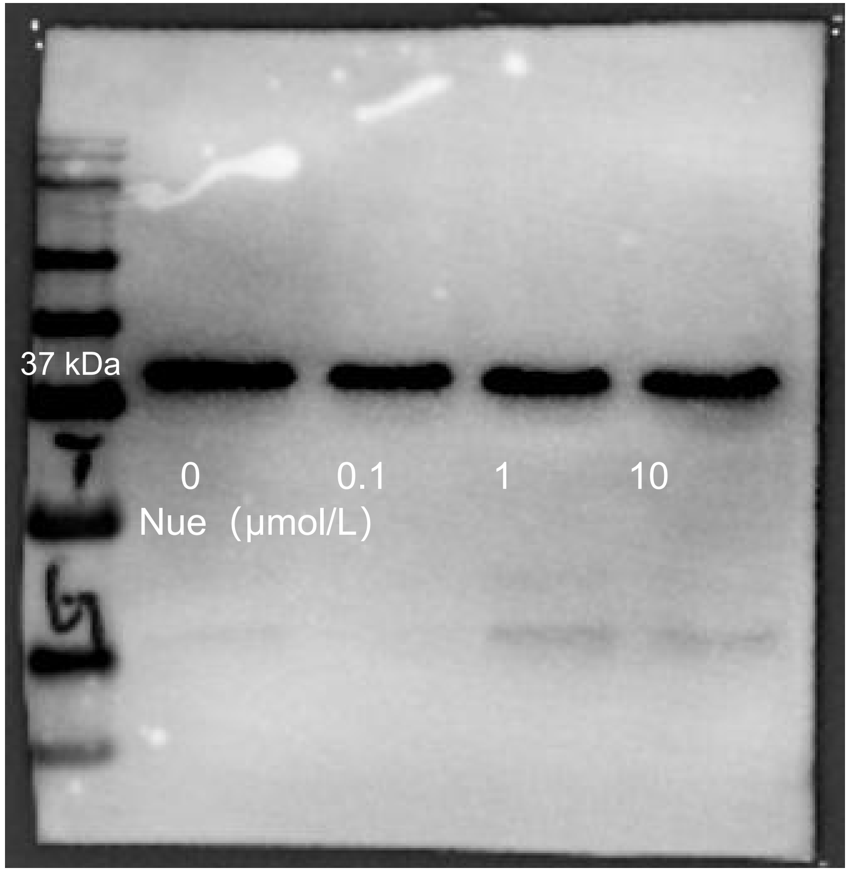

Figure 3E-BMP2

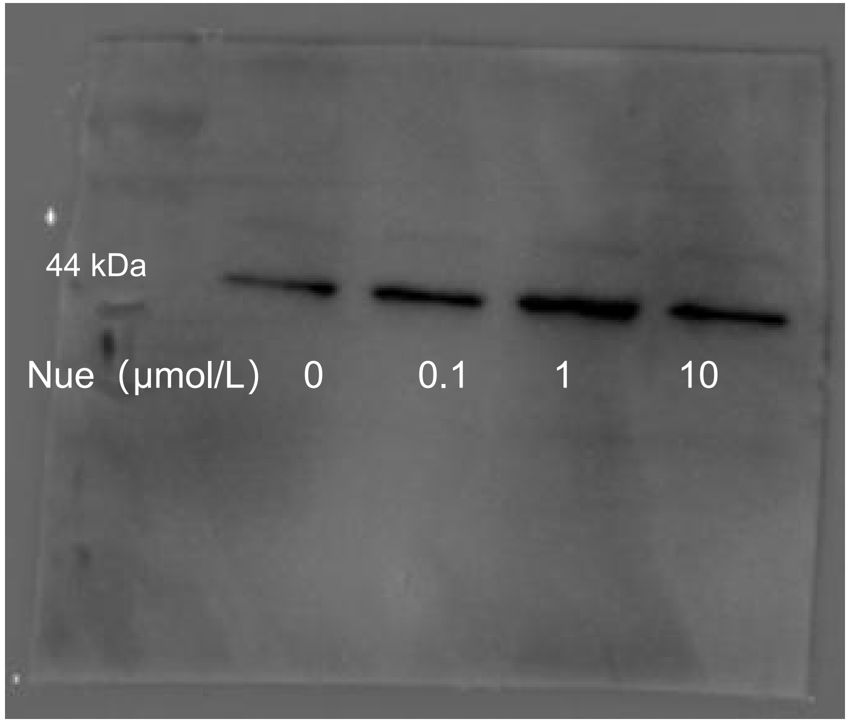

Figure 4A-Nue

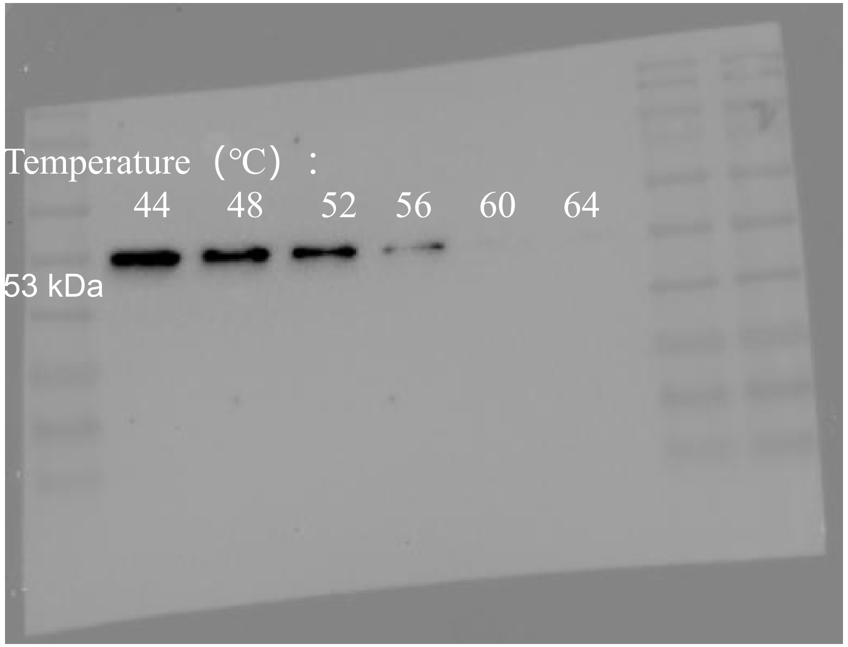

Figure 4A-DMSO

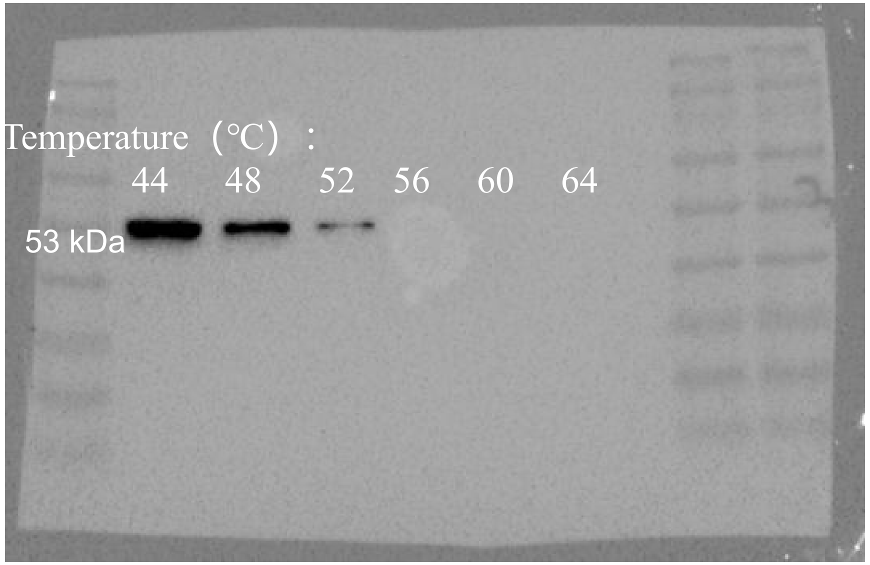

Figure 4B-TFEB

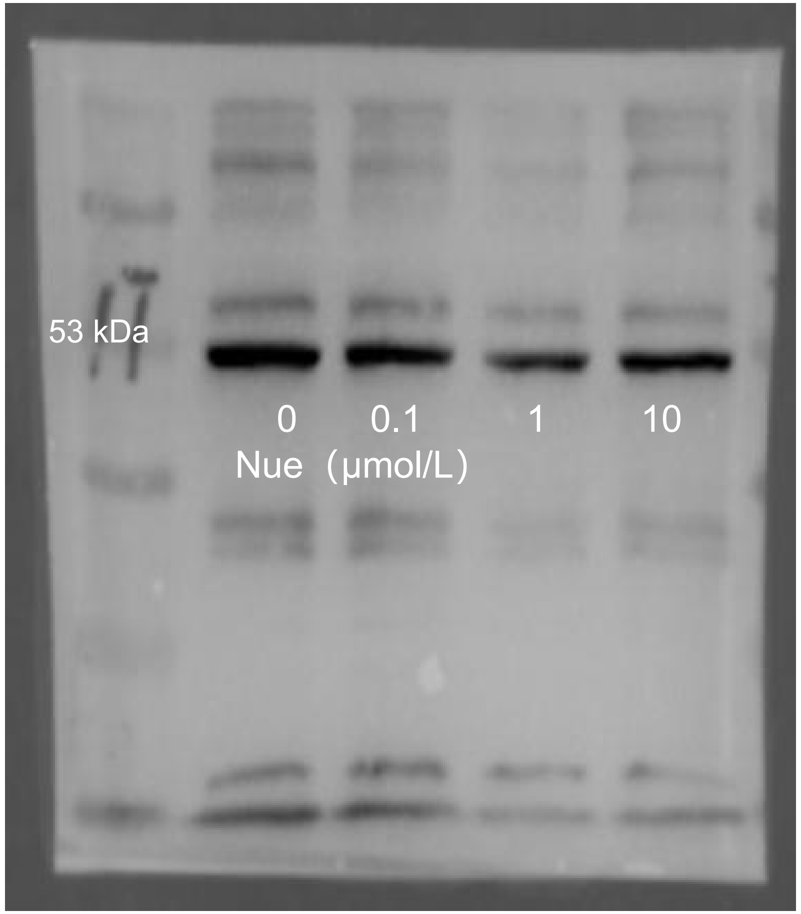

Figure 4B-α-Tublin

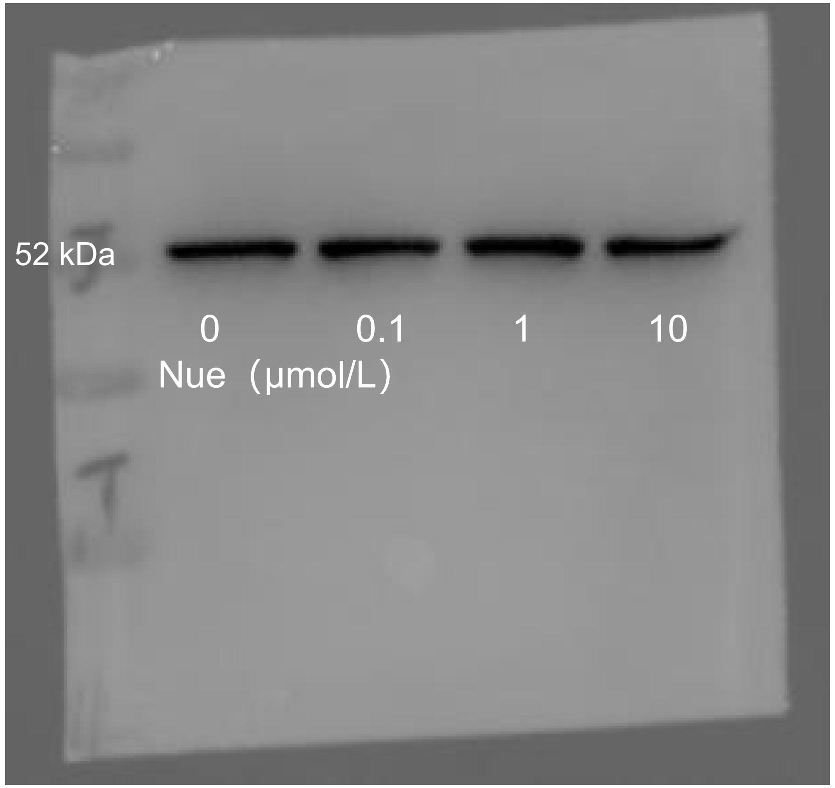

Figure 4B-TFEB

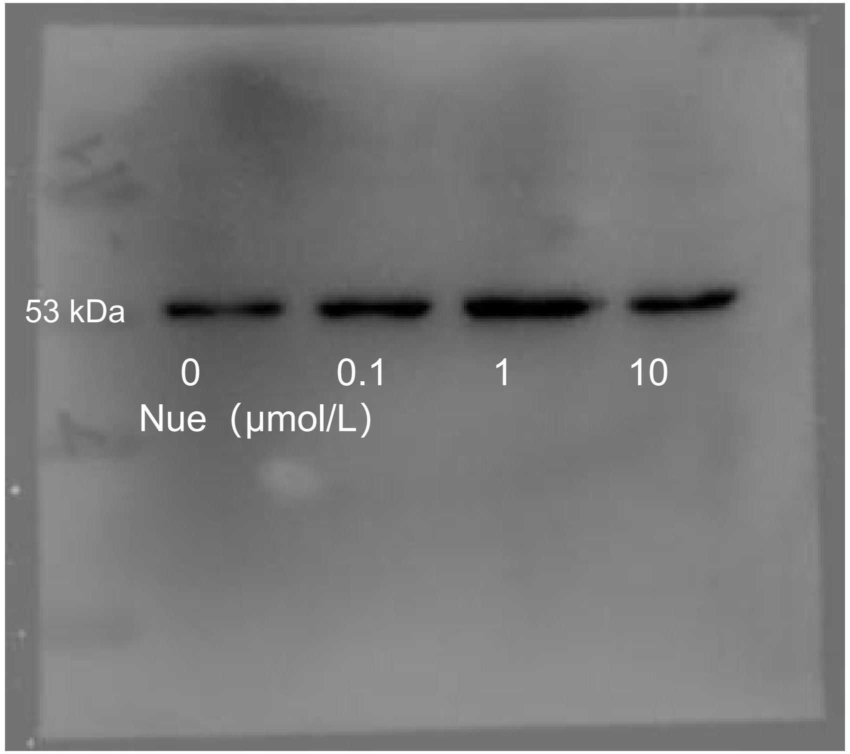

Figure 4B-H3

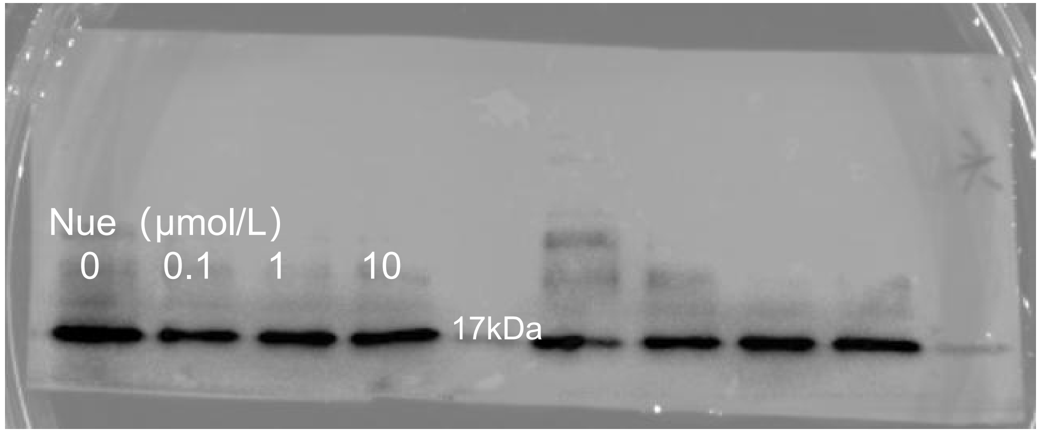

Figure 4G-TFEB

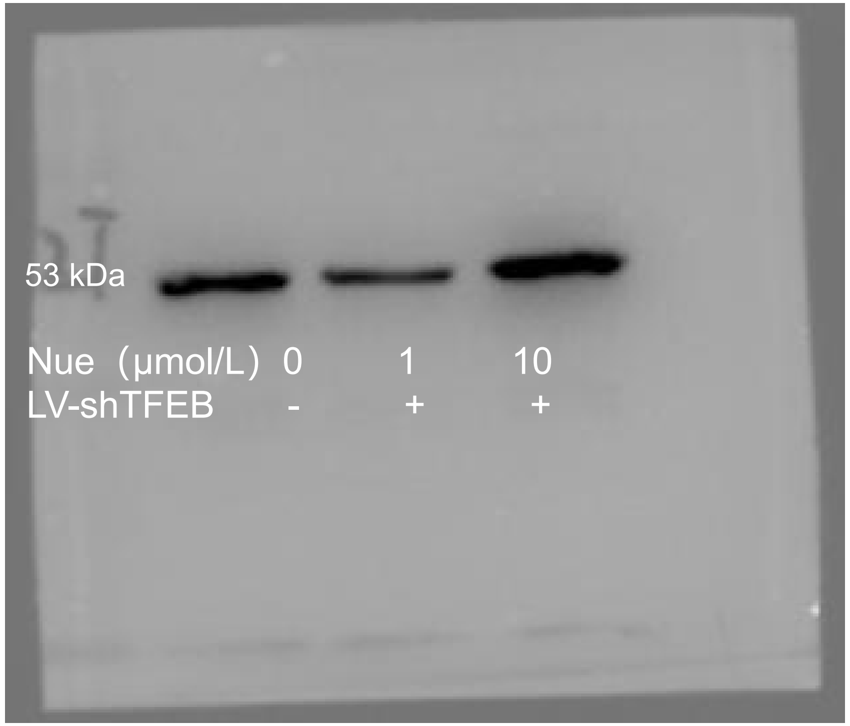

Figure 4G-β-actin

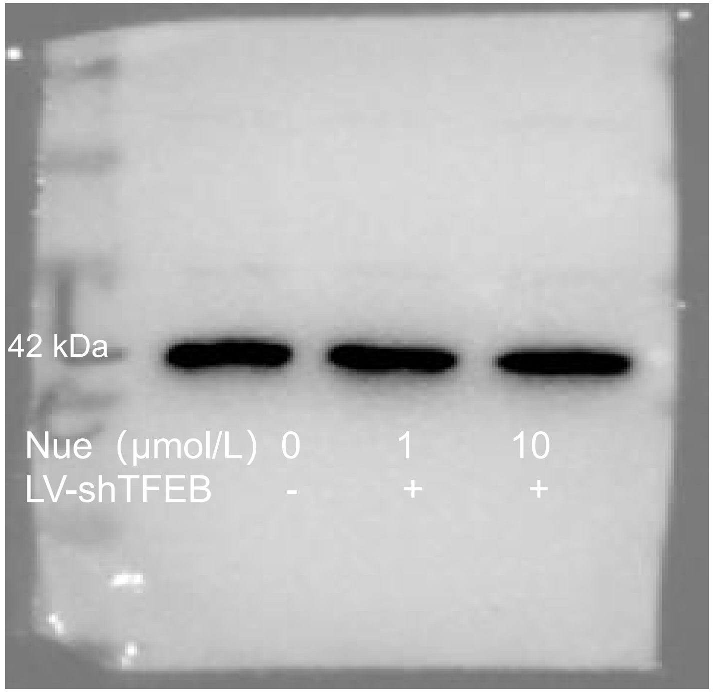

Figure 4K-ALP

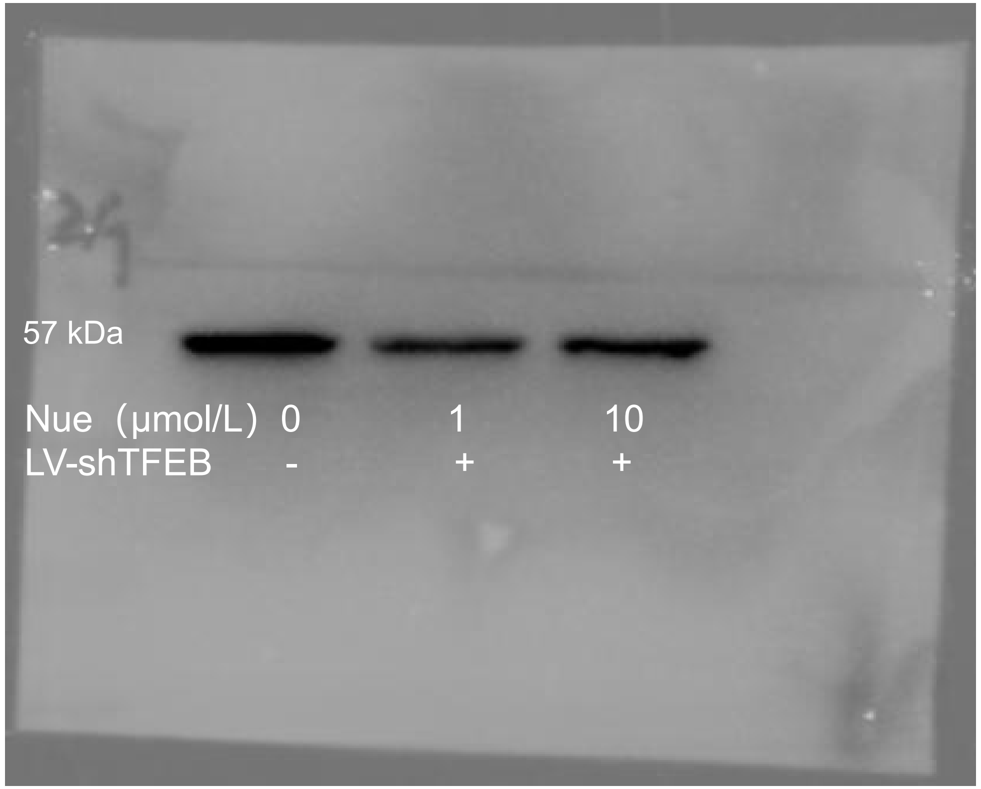

Figure 4K-RUNX2

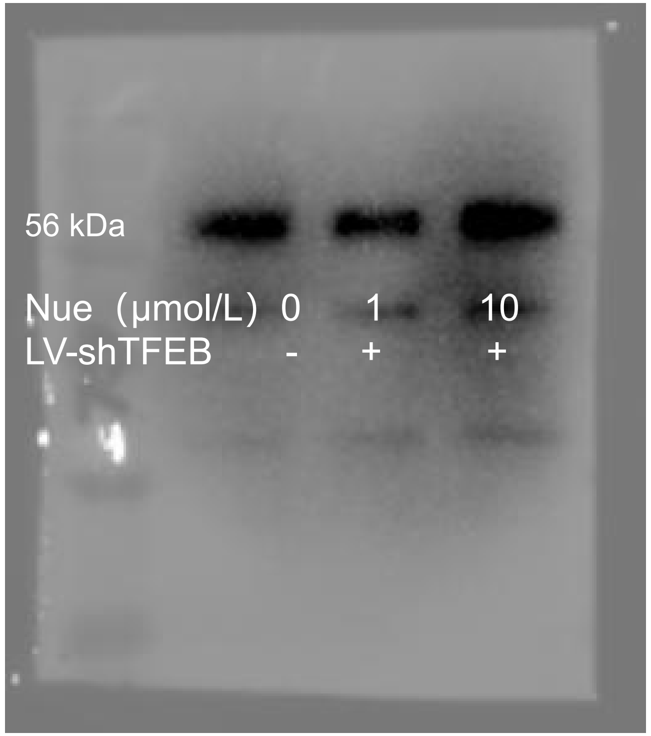

Figure 4K-BMP2

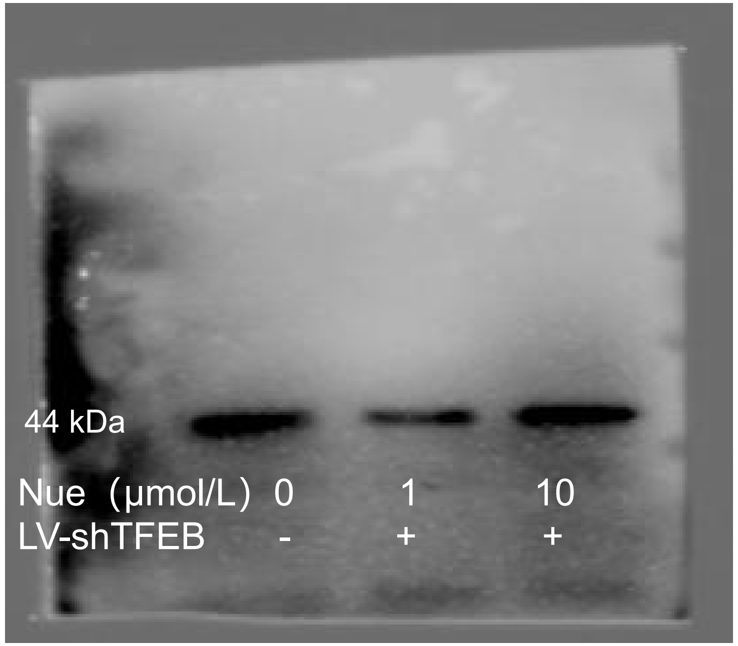

Figure 4K-GAPDH

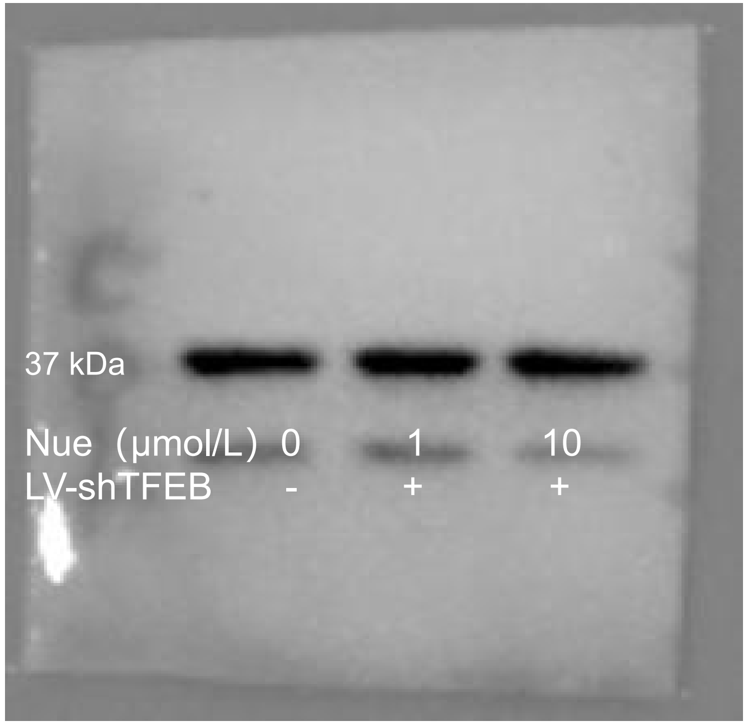

Figure 5A-LC3

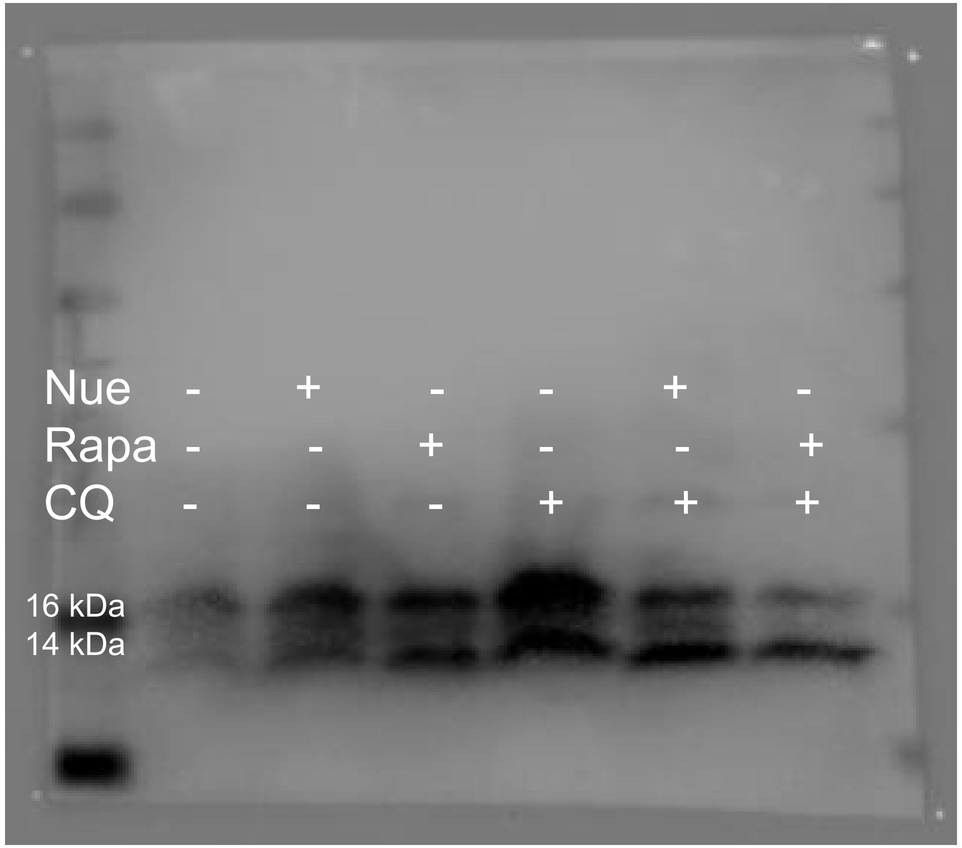

Figure 5A-P62

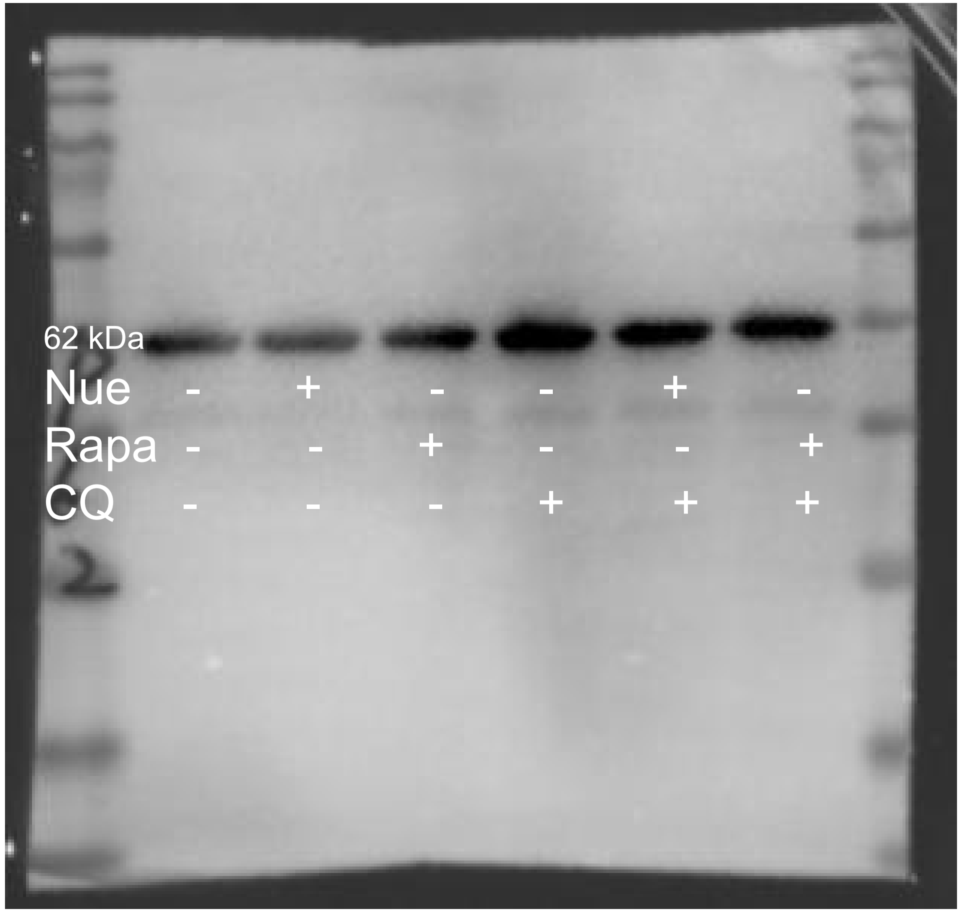

Figure 5A-GAPDH

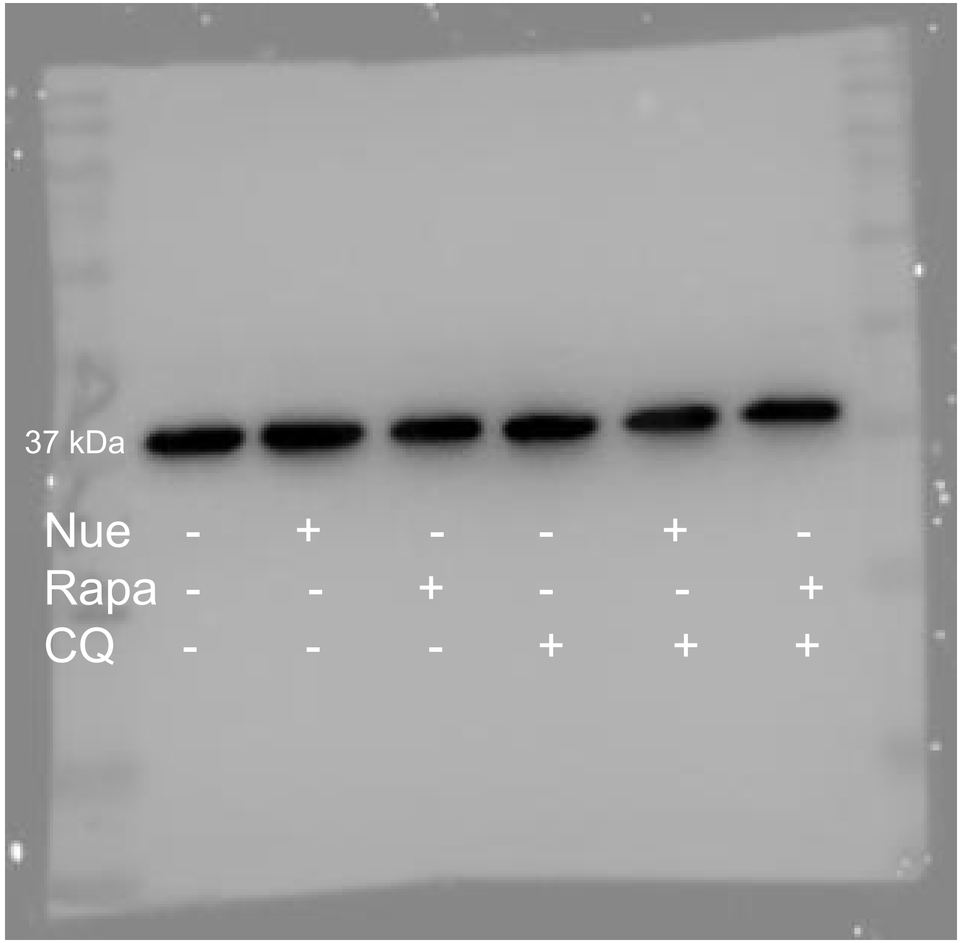

Figure 5E-ALP

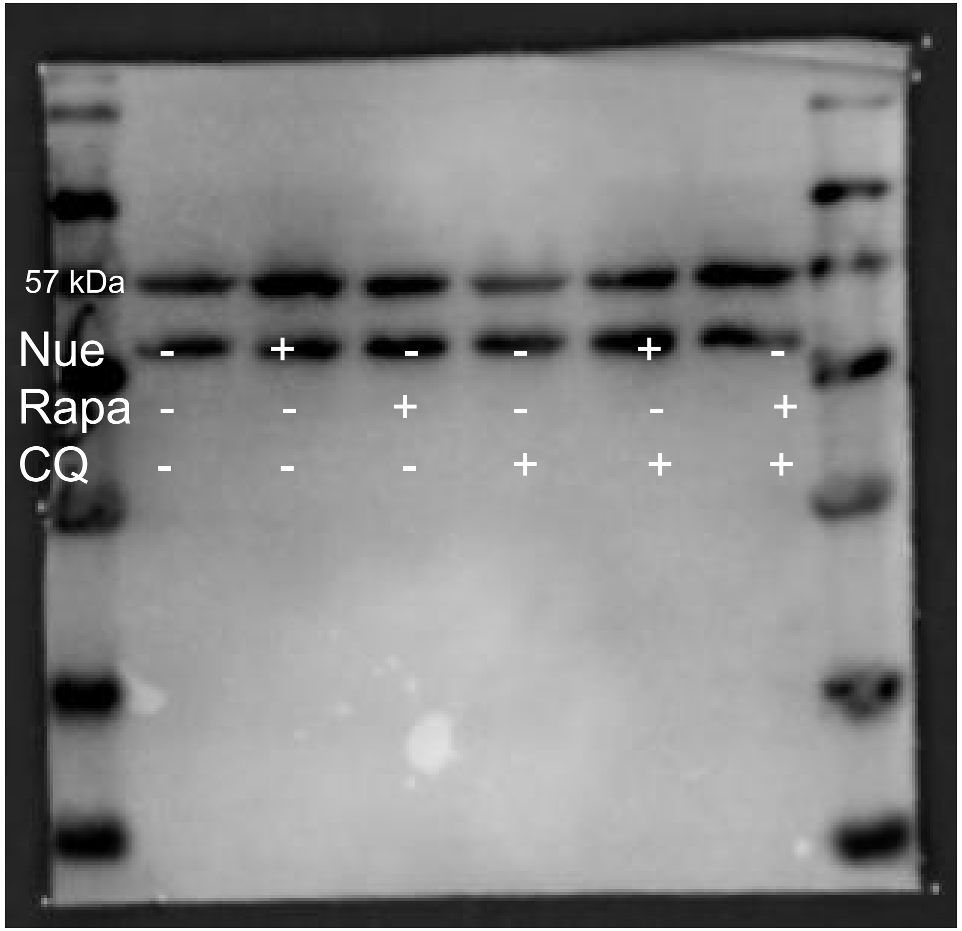

Figure 5E-RUNX2

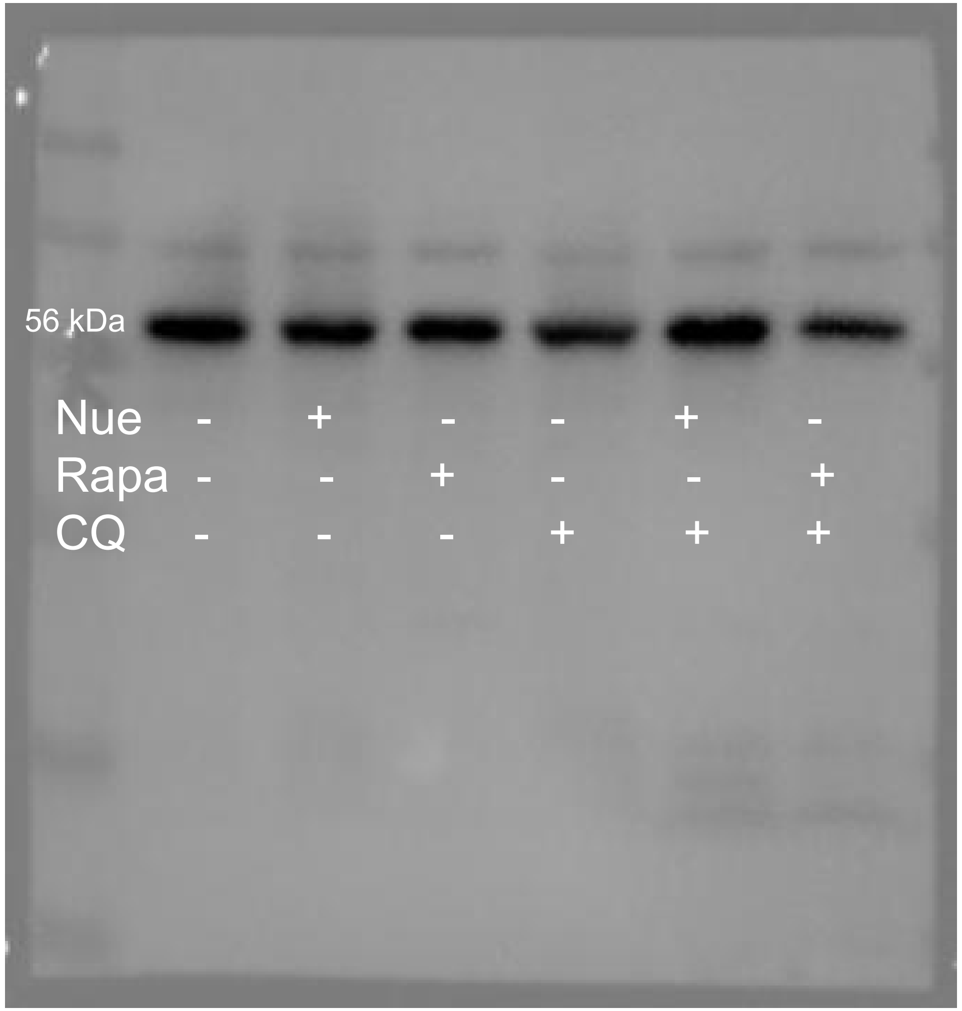

Figure 5E-GAPDH

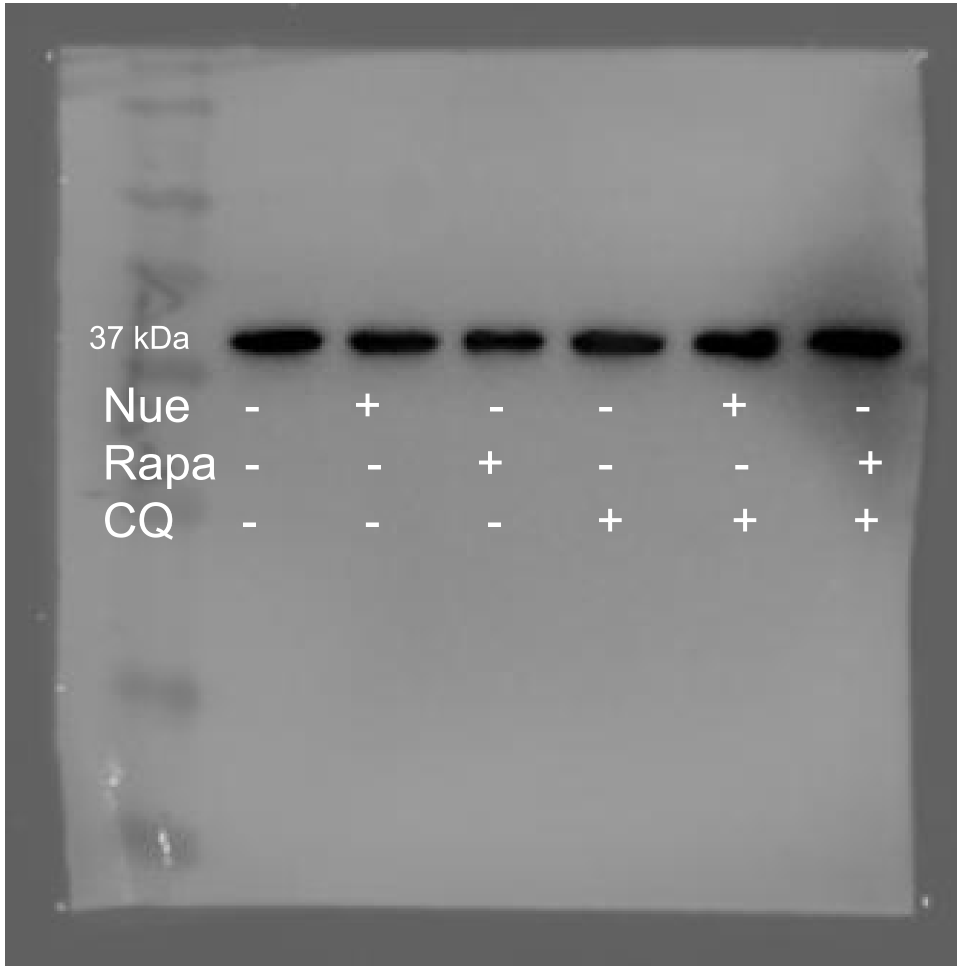

Supplement: Supplementary file 1 [file biomolecules-16-01370-s001.zip › Original Western blot images.pdf]
