# Supplementary material for: Nuciferine Ameliorates Ovariectomy-Induced Bone Loss via TFEB-Mediated Autophagy-Lysosomal Pathway
Source: Biomolecules. 2026 Sep 21;16(9):1370. doi: 10.3390/biom16091370 (PMC13604095; doi:10.3390/biom16091370)
Supplement: Supplementary file 1 [file biomolecules-16-01370-s001.zip › Supplementary Information Table S.1.-Table S.2.pdf]

Supplementary Information

Table S.1. Antibodies and Reagents

Antibodies	Company	Catalog No.
TFEB	Affinity Biosciences	AF7015
Beclin1	Affinity Biosciences	AF5128
H3	Affinity Biosciences	BF9211
LC3	Affinity Biosciences	AF0016
OSX	HUABIO	HA722817
RUNX2	Cell Signaling Technology	12556
α-tubulin	Cell Signaling Technology	2125
Goat anti-rabbit IgG H&L (HRP)	Cell Signaling Technology	ab6721
Rabbit anti-mouse IgG H&L (HRP)	Cell Signaling Technology	ab6728
p62	Abcam	RM1079
ALP	Abcam	ab307726
BMP2	Abcam	ab284387
GAPDH	X-Blot	XAb-G
β-actin	X-Blot	XAb-As
Alizarin Red S	Solarbio	G1452
Saturated Oil Red O staining solution	Solarbio	G1260
The Alkaline Phosphatase Color Development Kit	Beyotime	C3250S
Minimum Essential Medium α (MEMα)	Gibco	12571063
Fetal bovine serum	AusGeneX	C0225
EDTA	Servicebio	G4021

Penicillin-streptomycin solution	Servicebio	G4003
TRIzol reagent	Invitrogen	AM9738
The cDNA reverse transcription kit	Accurate Biology (AG)	AG11623
RIPA lysis buffer	Beyotime	P0013B
The BCA protein assay kit	Thermo Fisher Scientific	23227
The CCK-8 kit	Dojindo	CK04
Carboxymethylcellulose sodium	Merck	21902

Table S.2. Sequences of the primers

Name	Primer sequence(5'-3')
BMP2	Forward：ACTCGAAATTCCCCGTGACC
	Reverse：CCACTTCCACCACGAATCCA
RUNX2	Forward：ACTTCCTGTGCTCGGTGCT
	Reverse：GACGGTTATGGTCAAGGTGA
COL1A1	Forward：ATCAGCCCAAACCCCAAGGAGA
	Reverse：CGCAGGAAGGTCAGCTGGATAG
ALP	Forward：AACGTGGCCAAGAACATCATCA
	Reverse：TGTCCATCTCCAGCCGTGTC
GAPDH	Forward：CATGGCCTTCCGTGTTCCTA
	Reverse：GCCTGCTTCACCACCTTCTT
